# Age-dependent Transcriptional and Circuit Alterations in the brain Underlie Post-Anesthesia Neurobehavioral Dysfunction

**DOI:** 10.14336/AD.2025.0596

**Published:** 2026-06-06

**Authors:** Yun Li, Cosar Uzun, Syed Taufiqul Islam, Balaji Krishnamachary, Hangnoh Lee, Zihui Wang, Hui Li, Shaolin Liu, Junfang Wu

**Affiliations:** ^1^Department of Anesthesiology and Center for Shock, Trauma and Anesthesiology Research (STAR), University of Maryland School of Medicine, Baltimore, MD, USA.; ^2^Center for Neurological Disease Research, Department of Physiology and Pharmacology, Department of Biomedical Sciences, University of Georgia College of Veterinary Medicine, Athens, GA, USA.; ^3^Department of Medicine, University of Maryland School of Medicine, Baltimore, MD, USA.

**Keywords:** Anesthesia/surgery, aging, bulk RNA sequencing, neuronal activity, behavioral function

## Abstract

With rising life expectancy, more elderly individuals are undergoing surgery, highlighting the need to understand postoperative neurological complications. Studies have linked general anesthesia (GA) to olfactory dysfunction (OD) and cognitive decline following operation (OP), though mechanisms remain unclear. Using aged C57BL/6 mice subjected to laparotomy and 2-hour isoflurane exposure, we assessed behavioral and molecular outcomes through functional tests and bulk RNA sequencing (RNAseq). We observed persistent OD in the weeks following GA/OP, along with reduced limb strength and motor coordination, indicative of increased frailty. While no immediate cognitive deficits were apparent, aged mice exhibited delayed cognitive impairments, including diminished learning and memory in Y-maze and novel object recognition tasks, as well as increased apathy in nest-building behavior. RNAseq of the olfactory bulb (OB) at 1-day post-GA/OP revealed upregulation of ubiquitin-dependent proteins and catabolic processes in the aged mice compared to sham group. In contrast, transcriptomic analysis of the hippocampus (HI) at 5 weeks after GA/OP showed more extensive molecular pathway changes, correlating with the delayed impairment of cognitive functions. Compared to young adult mice, aged GA/OP mice demonstrated upregulated expression of genes associated with innate immunity and reduced neurogenesis in the OB, along with increased gliogenesis and reduced RNA splicing activity in the HI. Additionally, *in vivo* electrophysiology recordings from the OB mitral cell layer revealed alterations in neuronal excitability and circuit disruptions after GA/OP. Taken together, our findings provided functional, molecular, and circuit-level insights into the age-dependent persistent olfactory impairment and delayed cognitive dysfunctions following general anesthesia and surgery.

## INTRODUCTION

General anesthesia (GA) is vital for many surgical operations (OP) and diagnostic procedures, but it can cause neurological side effects such as olfactory dysfunction (OD), postoperative delirium (POD), and postoperative cognitive dysfunction (POCD) [1, 2]. OD, observed in both clinical and preclinical studies, includes symptoms like anosmia, parosmia, and dysgeusia [3, 4]. POD, a common complication in older patients, is marked by confusion and inattention, typically occurring immediately after surgery and lasting up to a week. In contrast, POCD involves longer-term cognitive decline affecting memory and attention, often persisting for weeks or even years, particularly in geriatric individuals [4, 5]. Both POD and POCD are linked to higher mortality, longer hospital stays, and reduced quality of life [6-8]. OD is not only a symptom of postoperative neurological issues but also an early indicator of neurodegenerative diseases like Parkinson’s and Alzheimer’s (AD) [9-11]. Studies show that OD often precedes cognitive decline by decades [12, 13] and is worsened by GA in aged mice [6]. Notably, 43.3% of elderly patients showed OD three days post-surgery, with 16.7% developing POCD [4], highlighting a strong association between these conditions.

Although the specific mechanisms are still being debated, experimental studies have revealed profound differences in anesthesia-induced cognitive impairment between aged and younger mice, rooted in neuroinflammatory cascades, amyloidogenic processing, synaptic plasticity deficits, and age-specific vulnerabilities in calcium homeostasis [14-16]. Possible mechanisms for this correlation between OD and POCD include the spread of tauopathy and other pathological proteins from the olfactory bulb (OB) to different regions of the brain [10]. Other prevailing theories suggest that OD is linked to decreased functional connectivity between olfactory regions and downstream brain areas including limbic structures, potentially accelerating cognitive decline [9]. Over the years, studies have revealed many potential molecular pathways that contribute to the deteriorating cognitive conditions after GA/OP, which includes NLRP3-dependent production of IL-1β and activation of formyl peptide receptor in peripheral myeloid cells, along with lipocalin-2 production in neurons leading to activation of pro-inflammatory microglia [17-19]. Recent studies show that GA/OP induces age-dependent delirium-like behavior in 18-month-old mice, marked by neuroinflammation, synaptic and mitochondrial dysfunction, and unfolded protein response dysregulation [20, 21]. In an AD mouse model, GA/OP also accelerated memory impairment, potentially via increased tau phosphorylation in the hippocampus [22]. However, neuroinflammation has long been acknowledged as a complex mechanism, and a comprehensive analysis of transcriptional changes in the GA/OP brain of aged mice has yet to be performed. Examining transcriptomic profiles through RNA sequencing (RNAseq) helps us identify genes involved with pathological development, allowing us to map out novel signaling pathways and regulatory networks that can be linked to novel mechanisms of POCD. The high sensitivity of RNAseq can detect both abundant and rare transcripts, which in turn reflects the functional state of the tissue in question.

Mitral cells as the output neurons of the OB plays a crucial role in odor processing by synaptic integration of local circuits and transmitting olfactory signals to downstream higher brain regions including peri-hippocampal structures [23]. Glutamatergic mitral cells as OB primary projection neurons have single apical dendrites projecting to individual glomeruli to receive excitatory input from olfactory sensory neurons [24, 25] and establish dendrodendritic synapses with local inhibitory GABAergic interneurons [26-28]. GABA released from these interneurons provides feedback or feedforward inhibition of mitral cells or other projection neurons or interneurons via GABA_A_ receptors and presynaptic inhibition of the olfactory nerve terminals via GABA_B_ receptors to suppress olfactory sensory input [26, 29]. Additionally, each mitral cell has multiple lateral dendrites running in the external plexiform layer to form dendrodendritic synapses with granule cells and local interneurons [26, 30, 31]. This provides another level of inhibition of mitral cells and other neuron types. Thus, mitral cell excitability and local network output reflect the excitation and inhibition balance or functional outcome of complex OB neural networks. Moreover, physiological assessment of neural circuit operation after GA/OP can provide mechanistic insights into OB dysfunctions in olfactory signal processing and even cognitive impairment. In the present study, we leveraged all these powerful experimental approaches to investigate the mechanisms underlying OD and POCD in a GA/OP model of aged male C57BL/6 mice, aiming to simulate conditions relevant to the aging human population. We focused on molecular pathway alterations in the brain and OB network dynamics over a time course extending up to five weeks post-surgery.

## MATERIALS AND METHODS

### Mouse General Anesthesia and Operation (GA/OP)

All experimental protocols received approval from the Institutional Animal Care and Use Committee (IACUC) at the University of Maryland School of Medicine or the University of Georgia. Young adult (10-12 weeks, 2.5-3.0-month-old) and aged (18-25-month-old) C57BL/6 male mice were sourced from Charles River Laboratories through the National Institute on Aging (NIA). The mice were housed in a 12-hour light/dark environment with unrestricted access to food and water. General anesthesia was administered using 2% isoflurane delivered in 100% oxygen via a precision vaporizer. Anesthesia depth was monitored through respiratory parameters (rate and depth) and reflex responses (palpebral and pedal). Core body temperature was maintained using a heated pad throughout the procedure. Following induction, mice were transferred to a surgical station with a nose cone for continuous isoflurane delivery. A midline laparotomy was performed by making a 1.5 cm incision from the xiphoid process to 0.5 cm above the pubic symphysis, sequentially penetrating the skin, abdominal musculature, and peritoneum. Following surgery, the incision site was treated with 0.25% bupivacaine in sterile saline, then closed in layers using 5-0 monofilament nylon sutures. The surgical procedure lasted approximately 10 minutes, after which mice were returned to the anesthesia chamber to complete the 2-hour isoflurane exposure. During recovery, animals were placed on a temperature-regulated pad for 30-60 minutes to stabilize core temperature. Postoperative monitoring included intensive observation for 4 hours post-GA/OP and daily assessments thereafter. A separate cohort of mice was subjected to 2 hours isoflurane exposure without surgical procedure. Sham mice, which did not receive GA/OP, remained in their home cages with access to room air for 2 hours to better reflect clinical conditions. Blinding was not possible in these studies due to the visible abdominal wounds in the mice.

### Assessment of Neurological Function

Following GA/OP, a battery of neurological behavioral tests was conducted for up to 38 days (Supplementary Fig. 1). The researcher conducting the behavioral experiments was blinded to group assignments until all three rounds of assessment were completed.

Odor memory (OM) test: To assess olfactory learning and memory, mice were individually housed overnight and tested using a previously established method [32, 33]. Briefly, a clean, dry, cotton-tipped wooden applicator (6 inches long) was inserted through a hole in the cage lid, allowing the mouse to familiarize itself with the applicator for 30 minutes. A fresh applicator was used for each subject. Cinnamon powder (McCormick, 100 ng/ml) was dissolved in water and stored in tightly sealed vials when not in use. During the first trial (T1), the mouse was exposed to the novel odor stimulus. The cotton tip of the applicator was dipped into the odor solution for 2 seconds before being inserted through the cage lid to a depth of approximately 2.5 cm. The total time the mouse spent sniffing the tip during the 5-minute trial (only when oriented toward the tip with its nose within 2 cm) was recorded. At the end of the trial, the applicator was removed. After a 60-minute rest period, the second trial (T2) was conducted under the same conditions. The relative sniffing time ratio between trials—calculated as [T2/T1] ×100[T2/T1]\times 100—was used to assess memory retention. A reduced sniffing time during T2 indicated recognition and memory of the familiar odor.

Buried food (BF) test: To assess olfactory function, the BF test was conducted to evaluate the animal’s ability to detect and locate a familiar food item hidden beneath the bedding. We examined how fast the animal can find buried (invisible) food pellets by using odor as a search cue [33]. In brief, mice were individually housed with ad libitum access to water but were food-deprived for 24 hours to enhance their motivation for food search during the test. The night before testing, a mini cookie was placed in each subject’s cage for odor familiarization, and its consumption was checked the following morning to confirm palatability. On the test day, each mouse was placed in a clean test cage (46 cm L × 23.5 cm W × 20 cm H) containing 3 cm of fresh bedding for a 10-minute acclimation period before being returned to its home cage. A uniformly sized mini cookie (~1 g) was then buried 2-3 cm beneath the bedding in a random corner of the test cage. After smoothing the bedding surface, the mouse was reintroduced to the test cage, and the latency to locate and begin consuming the buried cookie was recorded. The test was terminated if the mouse failed to find the cookie within 15 minutes.

Nest building (NB) test: The NB test is a simple, non-invasive behavioral assay commonly used in rodent studies (especially mice) to assess well-being, cognitive function, and social/motivational behaviors [34, 35]. Each mouse was housed individually in a standard cage with wood chip bedding. A pre-weighed pressed cotton square (nestlet) was placed within each cage, ensuring no other nesting material was present. Mice were left undisturbed for at least 12 hours overnight. In the following morning, nest quality was evaluated using a standardized 5-point scale: 0 (untouched nestlet) to 5 (perfect dome-shaped nest). Any unused nesting material was weighed to provide an objective measure of nesting ability. Nests were photographed for later analysis. Poor NB performance may indicate impairments in executive function, motor planning, and cognitive flexibility, as well as reduced well-being and motivation.

Grip Strength (GS) test: This test in mice is designed to measure neuromuscular function, particularly forelimb and/or hindlimb strength using a digital grip strength meter (Ugo Basile) based on our established protocols [36, 37]. Briefly, the forelimb GS of both paws was assessed by placing the mouse on a mesh wire grid connected to the device’s force transducer. Once the mouse secured a firm grip, it was gently held by the tail and slowly pulled away from the grid. The maximum force exerted on the mesh wire grid was recorded, with each mouse undergoing an average of 10 daily trials. Final GS values were normalized to body weight for comparison between groups.

Rotarod (RT) test: Locomotor function was measured with the Rotarod (Harvard Apparatus) as described in previous studies [36, 37]. The Rotarod's acceleration settings were 4 to 40 rpm over 90 s, with each trial lasting a maximum of 300 s. The mouse was placed on the device before initiation, and its latency to fall off the accelerating Rotarod was recorded. Individual scores from five trials were averaged and used for comparison between groups.

Y maze (YM): This test was performed to evaluate the hippocampus-dependent spatial working memory in mice, as described previously [36, 38, 39]. The Y-maze apparatus (Stoelting Co.) is consisted of three arms of identical length and width (A, B, C). During the test, one arm was selected randomly as the starting position for all mice, and each mouse was placed in the maze to explore freely for 5 min. Arm entries were recorded, and an alternation was completed when the mouse enters three different arms consecutively. The percentage of alternation was calculated with the following equation: total alternations × 100/(total arm entries - 2). Mice that scored significantly higher than 50% (the chance level for choosing an unfamiliar arm) were considered to have functional spatial working memory.

Open Field (OF) test: Spontaneous motor activity was evaluated using the OF test in a dark room illuminated by red lighting. Each mouse was placed in a corner of the open-field chamber (22.5 cm × 22.5 cm), facing the wall, and allowed to explore freely for 5 minutes. The Any-maze tracking program recorded all activity and analyzed various parameters, including total distance traveled, average speed, immobile time, freezing time, and time spent in the inner zone.

Novel object recognition (NOR) test: To evaluate non-spatial recognition memory, mice underwent the NOR test which leverages the natural tendency of rodents to explore new or unfamiliar objects over familiar ones [36, 40]. On the first day, mice were allowed to habituate in the open field apparatus for a 5-min period. On the second day of testing, mice were placed in the same arena with two identical objects, the time spent exploring each object was recorded using ANY-maze software (Stoelting) until the total exploration time accumulated to 30s. On the third day, one of the familiar objects was switched out with a novel object. Like the second day, testing stopped after each mouse went through a sum of 30-s exploration time. For both experimental days, a maximum duration of 40 minutes was set, after which the mouse was removed. Since mice inherently prefer to explore novel objects, a preference for the novel object with an exploration time of more than 15 s was considered to have intact learning and memory skills.

### RNA Extraction and Bulk RNA Sequencing (RNAseq)

Following euthanasia 24 hours and 5 weeks after GA/OP, mice were perfused with 50 mL of ice-cold saline. Total RNA was isolated from the OB and hippocampus (HI) of both sham and GA/OP mice using a miRNeasy isolation kit (Cat# 217084, Qiagen). After RNA extraction, samples were sent to Novogene (Sacramento, CA) for mRNA library preparation (poly A enrichment) and sequenced as paired-end (150 bp) on a NovaSeq 6000 platform (Illumina, CA). We used STAR aligner version 2.7.5 [41] to map RNA-Seq reads to the mouse reference genome (GRCm39) with GENCODE gene annotation (version M33). We quantified gene and isoform expression at the transcripts per million (TPM) level using RSEM 1.3.3 [42]. We used DESeq2 version 1.42.1 [43] for the differential expression analysis, with false discovery rates (FDR) derived from the Benjamini-Hochberg method. Genes with an FDR of less than 0.05 were considered to be differentially expressed and used for downstream pathway enrichment analysis. Gene ontology analysis was performed with cluster Profiler 4.10.1 [44] using DESeq2 output.

### *In Vivo* Electrophysiological Recording and Data Analysis

Surgical preparations for *in vivo* electrophysiological recordings and data analysis were performed as previously described with modifications [27, 33]. Animals were anesthetized by 2% isoflurane. To prepare for the recordings from the mitral cell layer (MCL) on the medial side of each olfactory bulb of awake mice, the craniotomy was made on the dorsal skull with the following coordinates: 5.50 mm anterior from Bregma and 0.3 mm lateral from the midline. A metal head-plate with a 4.2-mm round opening (Models 1 and 10; Neurotar, Helsinki, Finland) was attached to the skull with Metabond. One week after surgery, mice were habituated to head fixation in head-fixed treadmill apparatus (Neurotar, Helsinki, Finland) for 15 min/day in 5 consecutive days. On post-surgery day 13, animals from the isoflurane groups were subject to general anesthesia by isoflurane at a starting concentration of 5% as induction followed by 2.5% as maintenance concentration with a 75 ml/min room airflow for 2 hours. The depth of anesthesia was confirmed by the loss of hind paw withdrawal reflex every 15 minutes.

To assess the short-term and long-term effects of GA and surgery, recordings were performed 24 hours and 7 days after the GA cessation, respectively. Spontaneous neuronal excitability and network activities were recorded as described previously [27, 33] but with a 32-channel neural probe (60 μm shank width, 15 μm shank thickness and 50 μm inter-channel distance) inserted into the MCL on the medial side of each OB. To verify the position of the recording sites, probes were coated with two different dyes: red [Red-Dil-DilC18(3)] for the left OB and green [DiO-DiOC18(3), Invitrogen] for the right OB. Electrophysiological signals were detected by the neural probe and passed through a digital head stage to a 32-channel amplifier (Plexon DigiAmp, Plexon, TX, USA), where they were sampled at 1 kHz and bandpass filtered at 300-7,500 Hz for spikes and at 0.1-200 Hz for local field potential (LFP) before being sampled at 40 kHz by a Plexon Omniplex recording system (Plexon, Dallas, TX). Spikes were sorted from the raw data with Offline Sorter V4 software (Plexon). The separation of different units was performed by principal component analysis based on criteria of waveform parameters and multidimensional clusters. Both spike and LFP data were further analyzed with NeuroExplorer V5.204 (Nex Technologies) and graphed with Excel and Origin 2024. Instant spike frequency (ISF) calculated from inter-spike interval of individual units was utilized to assess neuronal excitability. At the population level, an average of ISF across multiple units was used to present each recording. A cumulative probability of ISF averaged across multiple animals in each group was applied to compare neuronal excitability among multiple groups. Power spectrum density of LFP signals ranging from 0.1 to 200 Hz, which covers different frequency bands including theta (0-12 Hz), beta (12-30 Hz), and gamma (low gamma, 30-60; high gamma 60-100 Hz), was measured to reflect oscillatory activities that are derived from interactions among excitatory and inhibitory neurons thus reflect network operation [33, 45, 46].

### Statistical Analysis

All quantitative data are displayed as individual data points in column graphs, presented as mean ± SEM. Statistical analyses were conducted using GraphPad Prism (version 9.5.0 for Windows, GraphPad Software; RRID: SCR_002798) for all tests. The Shapiro-Wilk test was used to assess data distribution normality. For behavioral analysis, the student’s t-test was used for data with normal distribution, while the Mann-Whitney U test was used for nonparametric data. For multiple group comparisons, one-way ANOVA was performed, followed by Tukey’s or Newman-Keuls post hoc tests for parametric data (when normality and equal variance assumptions were met). Detailed statistical analyses for each assay are provided in the figure legends, with a significance threshold set at p ≤ 0.05.

**Figure 1.**
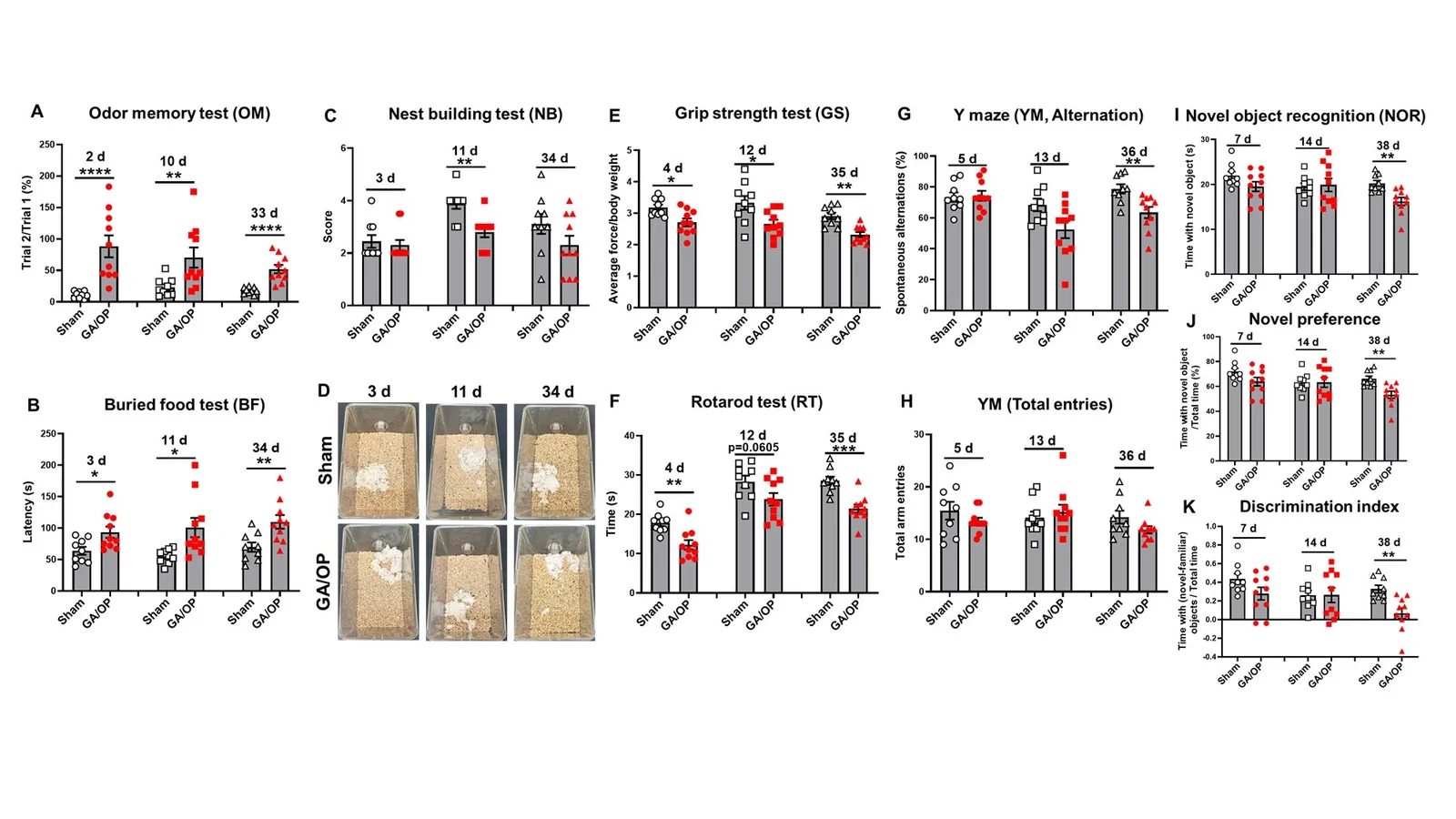
**General anesthesia (GA) and surgical operation (OP) in aged mice result in long-lasting neurological impairments**. (**A-B**) Olfactory dysfunction in GA/OP mice was demonstrated using the odor memory (OM, A) and buried food (BF, B) tests. (**C-D**) General well-being in aged GA/OP mice was impaired, as evidenced by the nest building (NB) test, with quantified nest scores (C) and representative images provided (D). (**E-F**) GA/OP mice exhibited impaired neuromuscular and motor functions, as demonstrated by reduced performance in the grip strength (GS, E) and rotarod (RT, F) tests. (**G-H**) Spatial memory was assessed using the Y-maze test, which revealed a significant impairment in spontaneous alternations at day 35 (G), while total arm entries remained unchanged (H). (**I-K**) Non-spatial memory was impaired at week 5 post-GA/OP, as assessed using the Novel Object Recognition (NOR) test. During the choice phase, the time spent exploring the novel object (I), the animal’s preference for the novel object (J), and the discrimination index (K) were recorded. n=9-10 mice/group. * p< 0.05, ** p< 0.01, *** p< 0.001, and **** p< 0.0001. Data was analyzed with Mann Whitney U-test for nonparametric data (A,C,E,G,J,K) and t-test in (B,F,H,I).

Data from *in vivo* electrophysiology experiments are presented as mean ± SEM. Our high-density (32 channels) neural probes enabled powerful assessment of network operation and neuronal excitability at the population level since each recording collected data from at least 30 individual neurons. After histological verification of probe position in the olfactory bulb, only data from on-target recordings were used for analysis. As the text in corresponding result sections explicitly described, our data collected from at least 7-11 recordings in 3-5 animals in each group after exclusion of off-target recordings were used for statistical analysis and significance calculation. This corresponds to at least 7 x 3 x 32 = 672 cells per group, more than the required sample size calculated by power analysis. Statistical analysis was performed using the OriginPro 2020 software (OriginLab Corporation, Northampton, MA). Comparison between the two groups was analyzed using the Mann-Whitney test. p - 0.05 was considered statistically significant.

**Figure 2.**
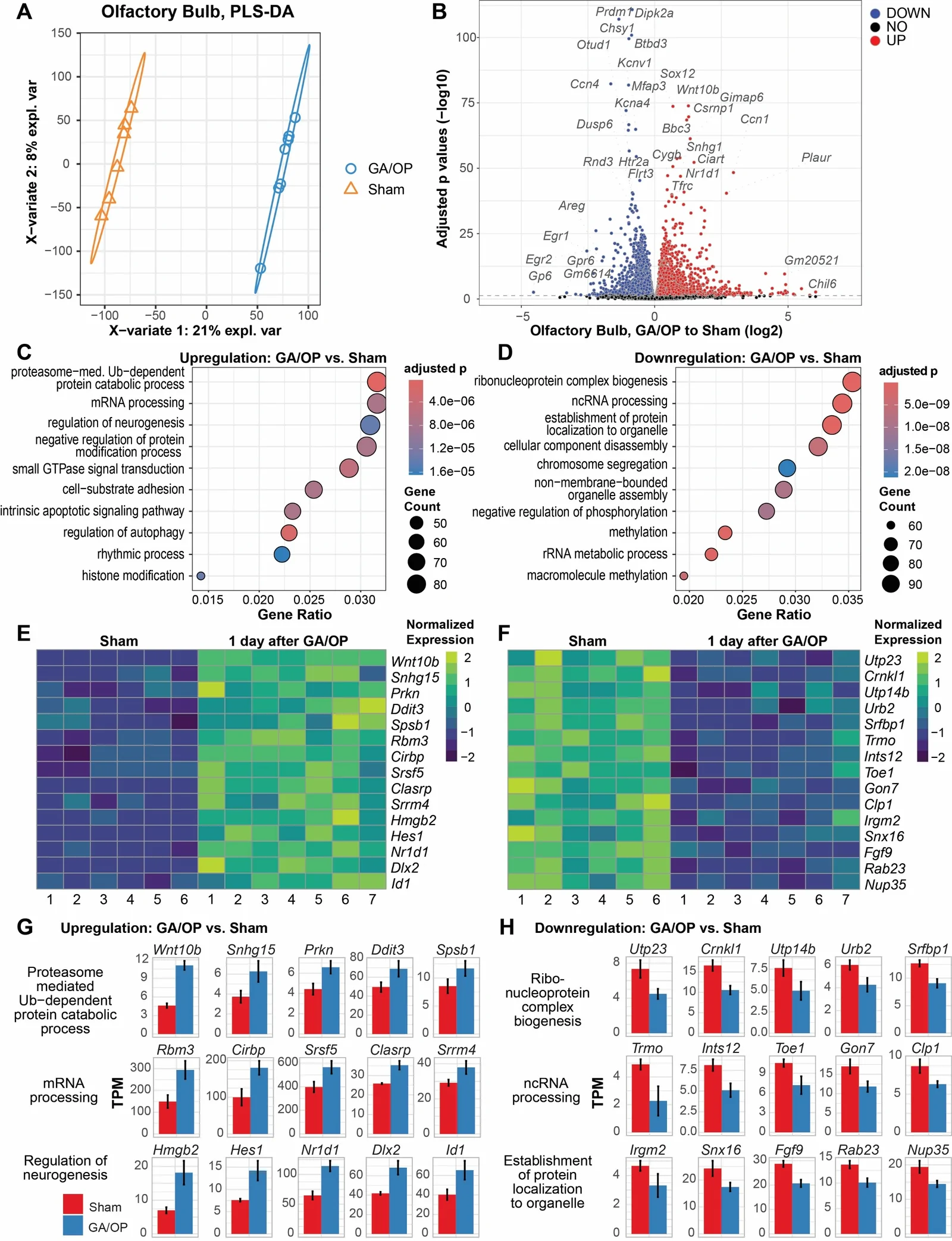
**The transcriptomic signature of olfactory bulbs in aged mice shows robust changes 24 hours after GA/OP cessation**. (**A**) PLS-DA plot for all normalized transcriptome genes is depicted for Sham and GA/OP groups. (**B**) Volcano plot displaying differentially expressed genes (DEGs) in the GA/OP versus Sham comparison. (**C-D**) Pathway enrichment analysis of up (C) and downregulated (D) DEGs with Gene Ontology molecular processes. (**E-F**) Heatmaps displaying DEGs associated with the top three upregulated pathways (E) and top three downregulated pathways (F) identified between GA/OP and Sham groups. Z-score normalization was applied to gene expression values. Heatmaps illustrate sample-wise variation (columns) across genes (rows). Color intensity reflects relative transcript abundance across samples: warmer colors for higher, cooler for lower. (**G-H**) Bar graph of genes within the top up- (G) and down-regulated (H) pathways that showed differential expressions. n=7 (GA/OP) and 6 (Sham) mice.

## RESULTS

### Exposure to anesthesia and surgery in aged mice leads to sustained neurological impairments

To investigate how short-term isoflurane exposure impacts neurological function in the aged mice, 20-month-old male C57BL/6 mice underwent abdominal surgery followed by a two-hour period under GA. A battery of behavioral tests was conducted to assess neurological functions up to 5 weeks after GA/OP (Supplementary Fig. 1). To examine the olfactory function, we performed odor memory and buried food test on the first, second and fifth week. The OM test aims to assess the ability of mice to identify and recall a sensory stimulus, which was reintroduced to them at least 1 hour after the 1^st^ trial. Due to cinnamon being a noxious stimulus to rodents, mice that can successfully differentiate and recall the odor would avoid the cotton tip and spend less time exploring it in the second trial. On day 2 and for many weeks after GA/OP, aged mice showed significant impairment compared to their sham group counterparts (Fig. 1A), showing a persistent presence of OD after GA/OP. Next, we examined the olfactory sensitivity of aged mice with BF test. After fasting for 24 hours before the test day, a cookie was buried beneath the bedding of the test cage and the latency time for each mouse to find the food was recorded. Similar to odor identification, the sensitivity of the GA/OP mice was significantly reduced compared to that of the sham mice (Fig. 1B). To elucidate the effects of GA alone, we subjected a separate group of mice to 2 hours isoflurane without abdominal surgery. In this group, mice initially didn’t show any impairments in the OM test but slowly developed worse sensory recall abilities on day 10 and 33 after GA/OP (Supplementary Fig. 2A), suggesting a delayed effect in disrupting the mice’s sense of smell. In BF tests, a portion of the aged mice fared worse than sham group on day 3, which quickly grew in numbers and became statistically significant on day 11 but recovered on day 34 (Supplementary Fig. 2B), indicating that many mice recovered their olfactory sensitivity in chronic periods. Taken together, these results suggest that both GA/OP and GA alone contributed to anosmia and altered perceptions of smell in aged mice.

To evaluate the general well-being and apathy-like behavior of aged mice, the nest building test was performed on days 3, 11, and 34 after GA/OP. During the first week after GA/OP cessation, aged mice showed no differences between Sham and GA/OP groups (Fig. 1C). At 11 d, however, most mice in the GA/OP group were unable to finish shredding the cotton nestlets overnight, and their nests scored predominantly poorer than their sham group counterparts (Fig. 1C-D). On day 34, two out of the ten mice that received GA/OP didn’t touch their cotton nestlets at all overnight, suggesting that some mice experienced progressively worsening of general well-being and increased symptoms of apathy. However, mice that were only exposed to GA without surgery showed no differences in NB test (Supplementary Fig. 2C).

Next, we examined the fore-arm neuromuscular and locomotor functions of the mice with grip strength, rotarod test, as well as open field test for spontaneous activity. On day 4, the GA/OP mice performed significantly worse in GS (Fig. 1E), reflecting increased frailty. On the other hand, mice that were only exposed to GA without surgery showed no differences in GS (Supplementary Fig. 2D). Still, four out of the ten mice in the GA group performed significantly worse than the sham group average on RT (Supplementary Fig. 2E), highlighting the vulnerability of aged individuals to volatile anesthetics. In later time points after surgery, GA/OP mice continued to show impaired neuromuscular functions in GS tests, suggesting that the increased frailty is a prolonged and systemic change. In terms of locomotor function, aged GA/OP consistently performed worse than sham mice on the rotarod from 4 to 35 d after surgery, further indicating that aged mice experience increased frailty (Fig. 1F). However, mice that only received GA showed no locomotor deficits immediately after exposure but had significantly shorter average latency times at 12 d, before making full recovery at day 35 (Supplementary Fig. 2E). Despite the significant impairment present in RT, OF tests revealed no differences in spontaneous activity between sham and either GA/OP or GA exposure groups in all three rounds of behavior experiments performed (Supplementary Fig. 3).

Finally, we examined cognitive functions with the Y-maze and Novel object recognition tests. Using YM, we found no differences in hippocampal-dependent spatial learning and memory in the first week following GA/OP (Fig. 1G-H). At 13 d after surgery, four out of the ten aged mice who underwent GA/OP started to show impairment in YM task. However, Mann-Whitney t-test revealed no statistical difference between groups. Furthermore, at 35 d after GA/OP, aged mice showed a drastic decrease in spontaneous alternations. The NOR was used to examine non-spatial recognition memory of mice (Fig. 1I-K). Similar as the observation in the YM, some mice started to show impaired performance in the NOR task during the first two weeks post-GA/OP, but t-test results between groups yielded no statistical differences. Most mice showed deficits in week 5 after GA/OP, signifying a delayed effect of the post-GA/OP stress on learning and memory functions. This phenomenon was further elucidated by examining the novel preference and discrimination indexes of each mouse (Fig. 1J-K). On the other hand, aged mice that only received 2h GA exposure didn’t show any symptoms of cognitive impairment during the first of the two weeks but displayed deficits in week 5 post-GA cessation in these tests (Supplementary Fig. 2F-K). During the training (sample) phase, sham, GA/OP, or GA alone mice spent equal time with the two identical objects (Supplementary Fig. 4A-C), indicating intact memory. There were no differences in mice body weight at 35 d post-operative stress among four groups (Supplementary Fig. 4D). Overall, these results indicate that exposure to anesthesia and/or surgery in aged mice leads to delayed cognitive impairment.

**Figure 3.**
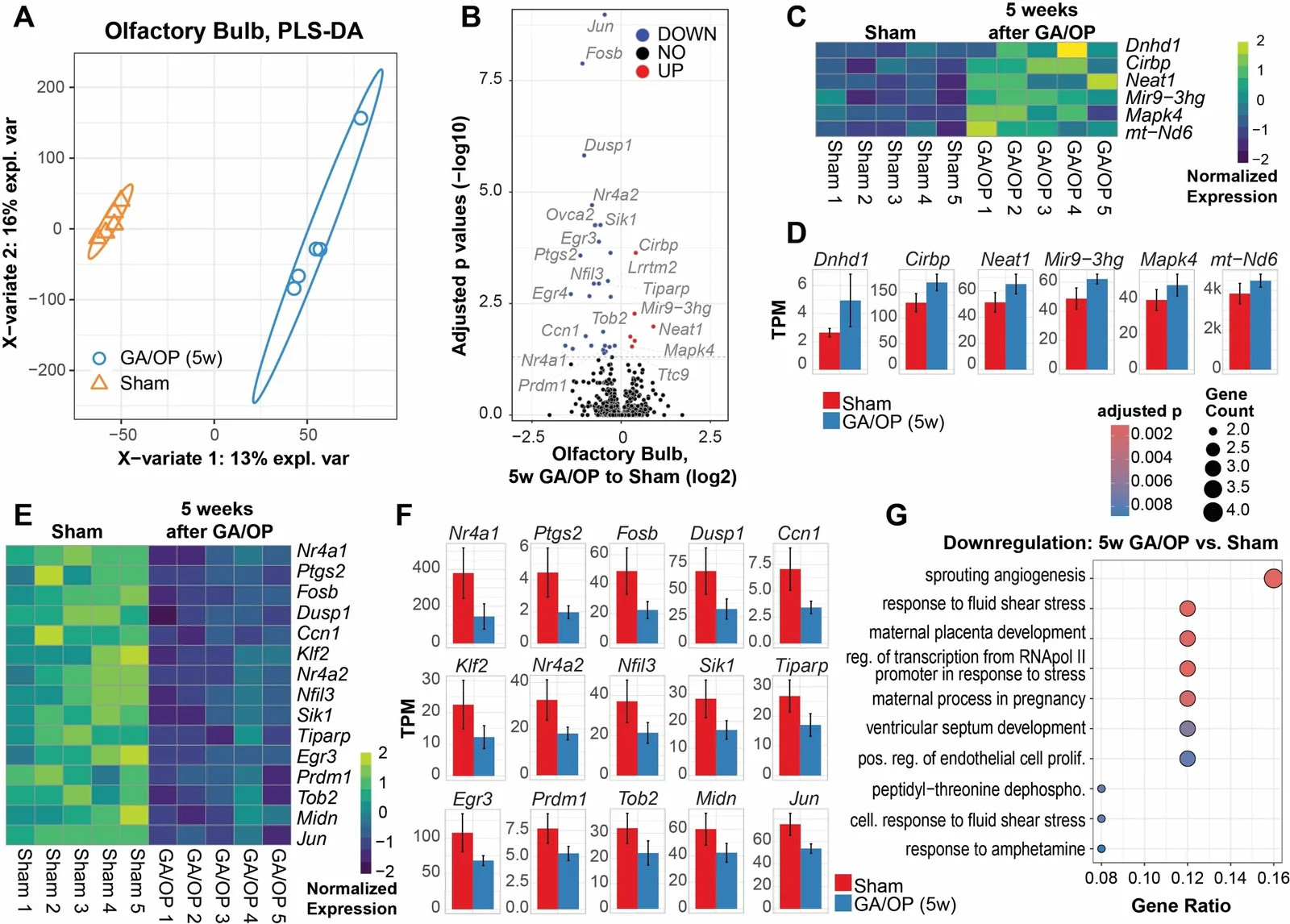
**RNAseq reveals molecular alterations in the olfactory bulb (OB) five weeks after GA/OP cessation**. (**A**) PLS-DA plot for all normalized transcriptome genes shows sample clustering by groups. (**B**) Volcano plot of all genes after pairwise comparison of aged GA/OP vs. aged Sham. (**C-D**) Heatmap and bar graph of all upregulated genes. Gene expression values were standardized as Z-scores. Columns represent individual biological samples, while each row corresponds to a gene. Higher expression levels are shown in warm colors, and lower levels in cool colors. (**E-F**) Heatmap and bar graph of all downregulated genes. (**G**) GO terms pathway enrichment analysis of downregulated DEGs. n=5 mice/group.

To determine whether the neurological impairments were age-related or induced by GA/OP, we treated a separate cohort of young adult mice (6-9 weeks old) with laparotomy and 2 hours of isoflurane. In the first week post-treatment, no deficits were observed in odor memory recall, olfactory sensitivity, or neuromuscular, locomotor, and cognitive functions compared to sham controls (Supplementary Fig. 5). These findings suggest that the behavioral deficits observed earlier are specific to aged (20-month-old) mice and primarily age-related.

**Figure 4.**
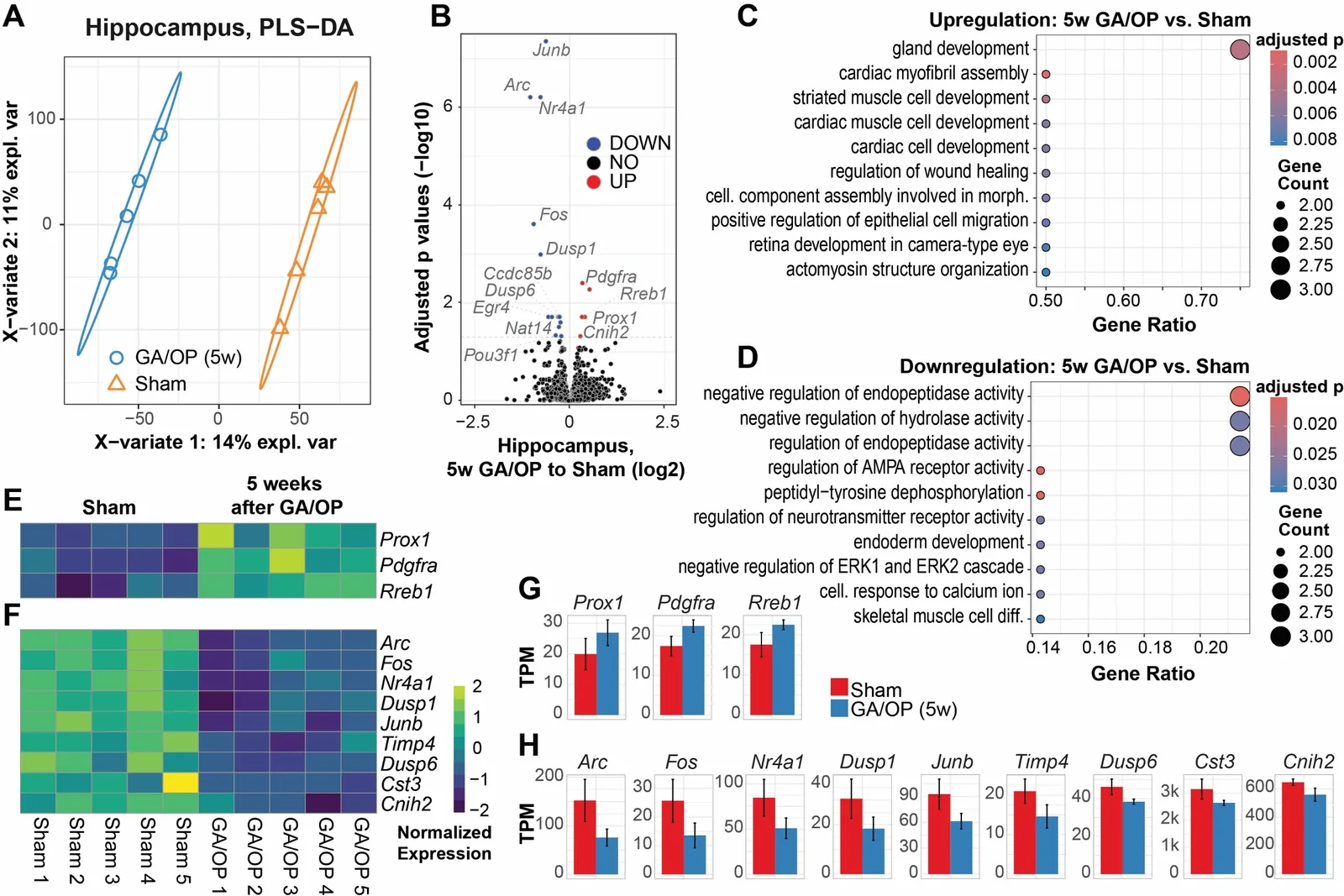
**GA/OP exposure in aged mice leads to sustained molecular changes in the hippocampus (HI) observed five weeks post-surgery**. (**A**) PLS-DA plot for all transcriptome data. (**B**) Volcano plot of genes in pairwise comparison of aged GA/OP vs. aged Sham. (**C-D**) GO terms pathway enrichment analysis of upregulated (C) and downregulated (D) DEGs. (**E-H**) Heatmap and bar graph of all up (E, G) and downregulated (F, H) genes. In the heatmaps, expression levels are normalized and represented as Z-scores. Rows indicate gene identity, and columns denote individual samples. Warmer colors indicate higher expression; cooler colors indicate lower expression. n=5 mice/group.

### Molecular pathways in the OB are altered 24h after GA/OP cessation

Given the olfactory impairments observed in GA/OP-treated mice, we examined molecular changes in the OB to better understand the pathogenesis of OD, using the GA/OP model to mimic the human condition. At 24h after GA/OP cessation, RNAseq was employed on OB tissues obtained from aged mice that underwent sham procedures or 2h GA/OP. PLS-DA of all normalized gene counts showed clear separation between the two groups (Fig. 2A). Further examination of differentially expressed genes (DEGs) with a false discovery rate (FDR) of less than 0.05 showed dramatic changes in GA/OP vs. Sham groups (Fig. 2B). Pathway enrichment analysis revealed “proteasome-mediated ubiquitin-dependent protein”, “catabolic process mRNA processing” and “regulation of neurogenesis” as the top three upregulated pathways (Fig. 2C), whereas “ribonucleoprotein complex biogenesis”, “ncRNA processing” and “establishment of protein localization to organelle” were the top downregulated pathways (Fig. 2D). Notable DEGs that GA/OP upregulated (Fig. 2E) includes *Wnt10b*, which is a main regulator of the Wnt pathway; *Snhg15*, which produces a long non-coding RNA that contributes to cell proliferation and acts as a sponge for microRNAs; *Prkn*, which mediates the production of Parkin, a molecule well-known for its role in Parkinson’s disease (PD); *Ddit3*, which regulates the DNA damage inducible transcript 3 molecule (CHOP) and can be induced by cellular stress. Moreover, several DEGs in the top three upregulated pathways are involved in RNA splicing (*Cirbp, Rbm11, Srsf5, Clasrp*) and protein modification (*Spsb1, Hes1, Nr1d1*), signifying a cascade of aberrant splicing patterns and post-translational modifications, which can be associated with diseases like neurodegenerative disorders and metabolic disorders. Amongst the DEGs that are part of the top three downregulated pathways (Fig. 2F), many are involved in mRNA splicing and binding activity (*Utp23, Crnkl1, Trmo, Ints12, Toe1, Gon7, Clp1*), while others play a role in regulating cell cycle (*Crnkl1, Utp14b, Urb2, Srfbp1*). For rigorous quantification, gene-level expression within each enriched pathway is depicted as bar graphs, with normalized transcript abundance (TPM) plotted on the y-axis to account for library size and sequencing depth (Supplementary Fig. 5). Subsequently, we interrogated the transcriptomic landscape of the HI region to elucidate early molecular perturbations that may prime or initiate downstream pathological cascades. PLS-DA of normalized gene expression profiles revealed distinct clustering between GA/OP and Sham groups. Differential expression analysis further identified pronounced transcriptional shifts in the GA/OP condition relative to Sham (Supplementary Fig. 6A). Although transcriptional changes in this region were relatively modest compared to the OB, as illustrated by the volcano plot (Supplementary Fig. 6B), pathway enrichment analysis revealed biologically intriguing signatures. The top enriched pathways upregulated by GA/OP were “regulation of membrane potential”, while the “generation of precursor metabolites and energy” pathway was downregulated (Supplementary Fig. 6C).

**Figure 5.**
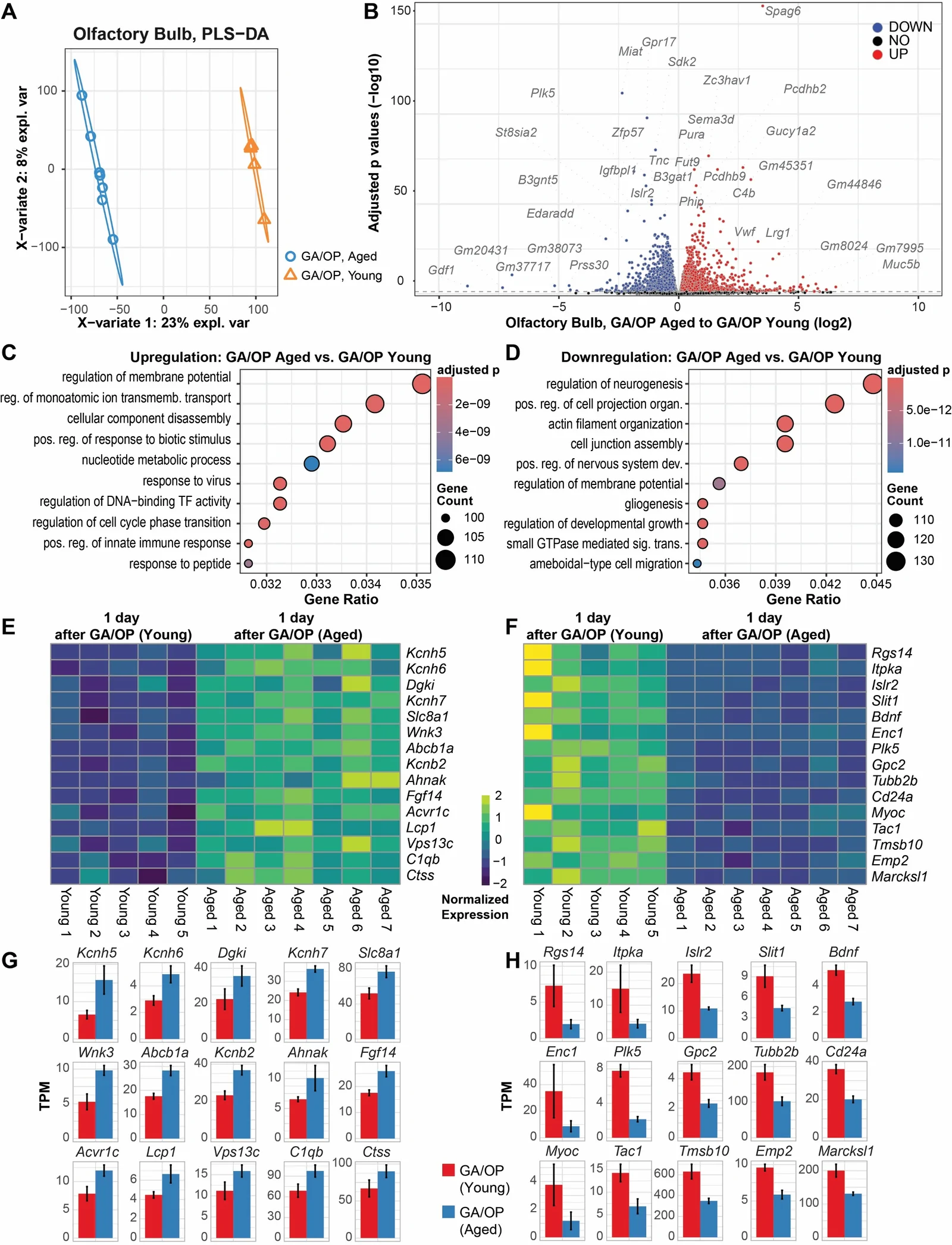
**Transcriptional changes in the olfactory bulb (OB) are compared between aged and young adult mice 24 hours after GA/OP cessation**. (**A**) PLS-DA plot for normalized transcriptome genes is depicted for young and aged GA/OP groups. (**B**) Volcano plot showing DEGs in a pairwise comparison of aged GA/OP vs. young GA/OP mice. (**C-D**) GO terms analysis of up (C) and downregulated (D) DEGs. (**E-H**) Heatmaps of DEGs from the top three upregulated (E) and downregulated (F) pathways showing relative transcript levels that have been normalized and represented as z-scores across samples (columns), along with bar graphs for quantification of individual genes (G-H). Warmer hues correspond to increased expression, whereas cooler hues denote reduced expression. n=5 (Young GA/OP) and 7 (Aged GA/OP) mice.

**Figure 6.**
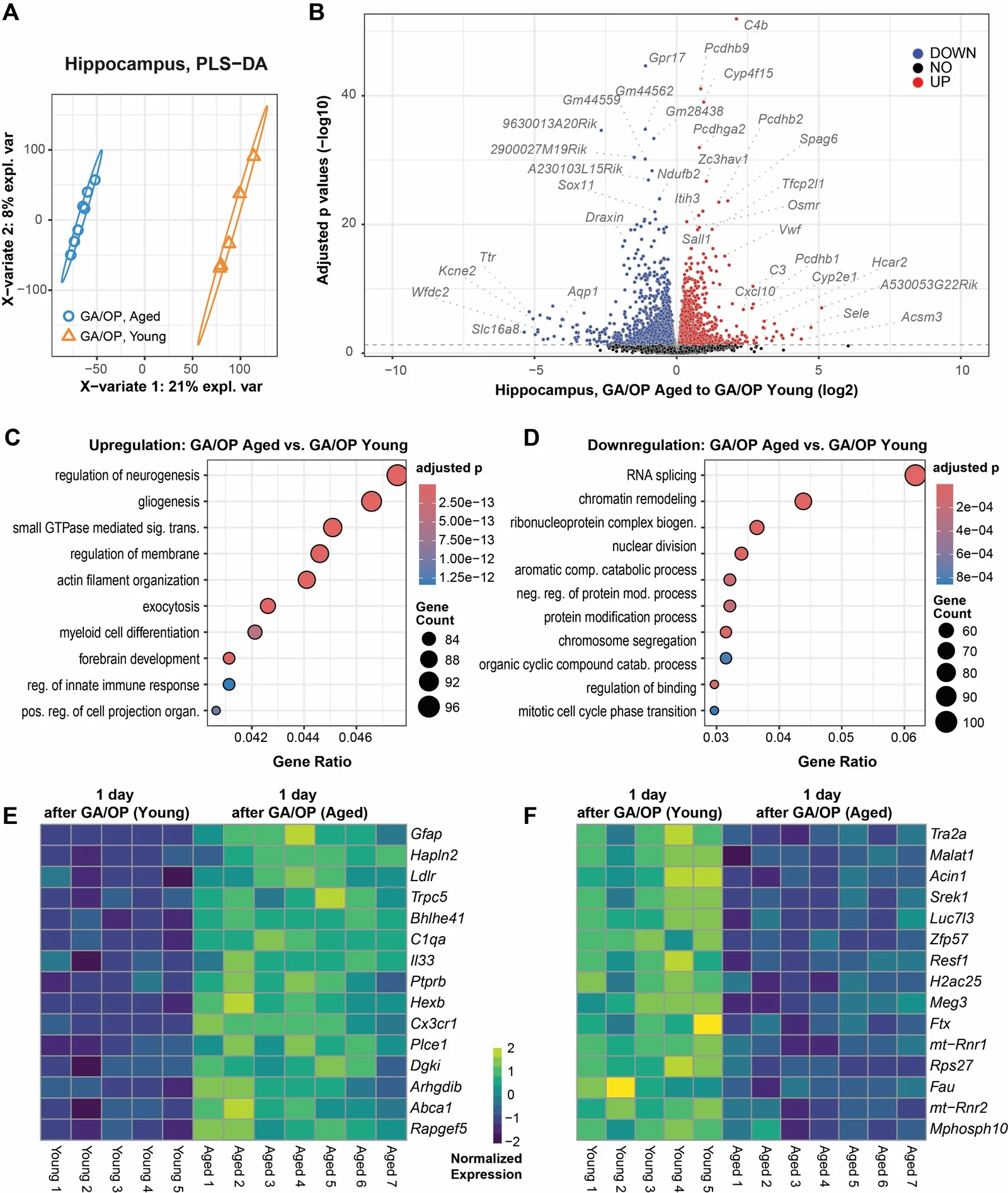
**Age-related effects on hippocampal (HI) transcriptomic signatures 24 hours after GA/OP cessation**. (**A**) PLS-DA plot for normalized transcriptome genes. (**B**) Volcano plot showing DEGs in Aged GA/OP vs. Young GA/OP groups. (**C-D**) GO terms analysis of up (C) and downregulated (D) DEGs. (**E-F**) Heatmap of DEGs associated with the top three upregulated pathways (E) and top three downregulated pathways (F). Expression levels are normalized and represented as Z-scores. Each column represents an individual sample, and rows correspond to individual genes. Warmer colors indicate higher expression; cooler colors indicate lower expression. n=5 (Young GA/OP) and 7 (Aged GA/OP) mice.

Notable DEGs of the top upregulated pathway (Supplementary Fig. 6D) include *Edn1* (endothelin-1), *Prkn* (Parkin), *Nr3c2* (mineralocorticoid receptor) and *Tmem108* (Transmembrane 108). In contrast, notable DEGs of the top upregulated pathway include *Aass* (Aminoadipate-semialdehyde synthase), *Il10rb* (Interleukin 10 receptor subunit β), *Coa6* (Cytochrome C oxidase assembly factor 6) and *Akt2* (AKT serine/threonine kinase 2). Gene-level expression of all DEGs in the top 3 up and downregulated pathways are also plotted as bar graphs with TPM for further analysis (Supplementary Fig. 7). Taken together, these results suggest that aged mice exhibit molecular pathway alterations in the OB in response to GA/OP, including cellular stress, alternative RNA splicing, and post-translational protein modifications, which may contribute to post-operative OD.

**Figure 7.**
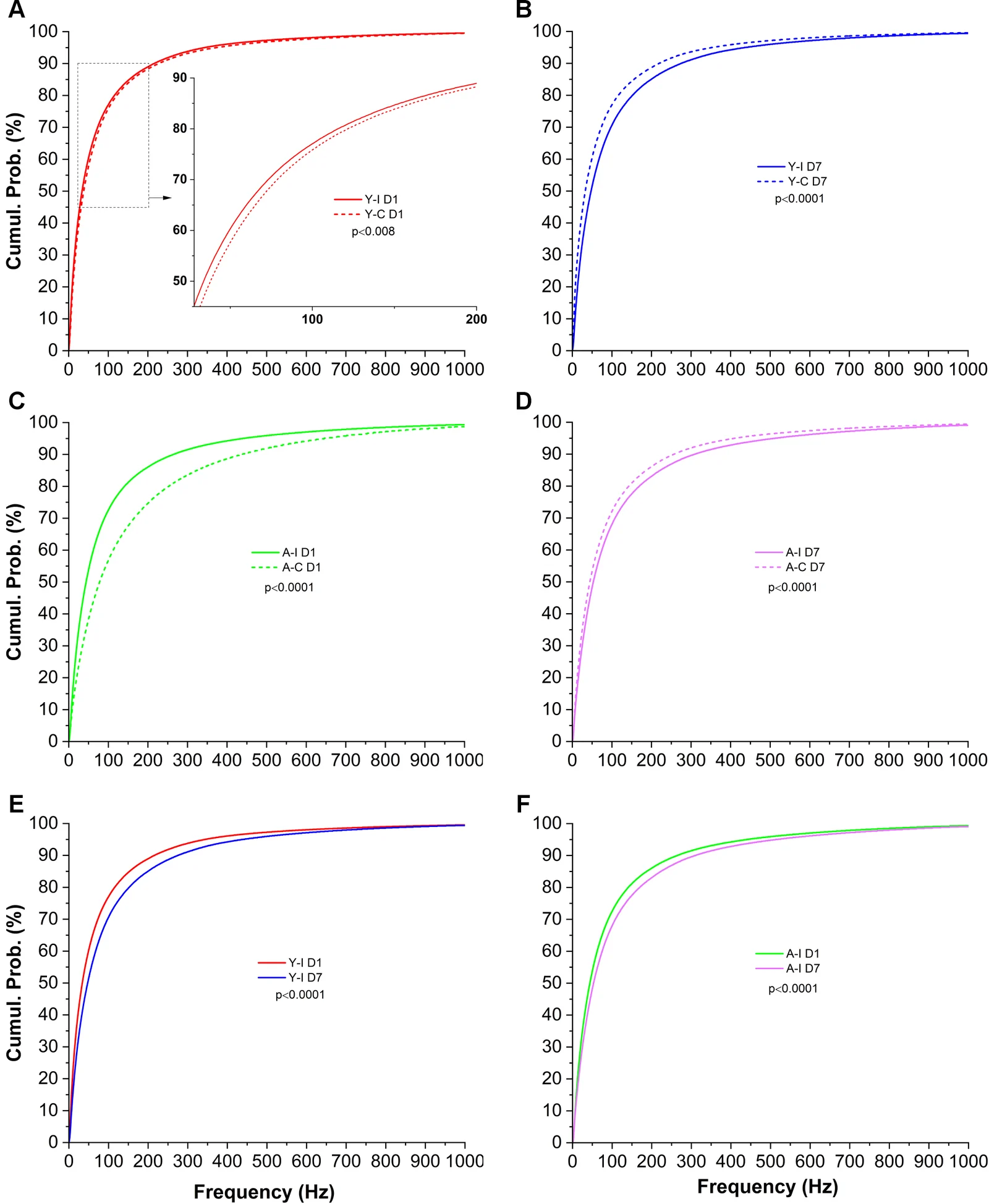
**Acute and chronic effects of isoflurane on neuronal excitability in the OB mitral cell layer**. (**A-D**) Comparison of the cumulative probability of spiking frequency between isoflurane-exposed and control groups on day-1 (A&C) or day-7 (B&D) post-isoflurane in young (A&B) or aged (C&D) animals. (**E-F**) Comparison of cumulative probability of spiking frequency between day 1 and day 7 post-isoflurane in young (E) or aged mice (F). Y-I D1: young post-isoflurane day 1 (n=5); Y-C D1: young control day 1 (n=5); Y-I D7: young post-isoflurane day 7 (n=5); Y-C D7: young control day-7 (n=5); A-I D1: aged post-isoflurane day-1 (n=4); A-C D1: aged-control day 1 (n=4); A-I D7: aged post-isoflurane day 7 (n=3); A-C D7: aged control day 7 (n=4). Comparison between the two groups was analyzed using the Mann-Whitney test.

### RNA sequencing identifies sustained molecular changes in the OB and HI regions 5 weeks after GA/OP cessation

To investigate the underlying mechanisms of sustained neurological impairments in aged mice following GA/OP, we performed RNAseq on OB and HI tissue samples collected five weeks post-operation. In the OB region, although PLS-DA showed clear separation between the two groups of sham and GA/OP (Fig. 3A), only a small number of DEGs were detectable, with most of them being downregulated genes (Fig. 3B). For the few upregulated DEGs (Fig. 3C-D), their functions include regulation of microtubule motor activity (*Dnhd1*), DNA damage and translational control (*Cirbp, Neat1, Mir9-3hg*), along with one of the mitogen-activated protein kinases (*Mapk4*) and the mt-Nd5 gene, which enables NADH dehydrogenase. For the downregulated DEGs (Fig. 3E-F), most of them are involved in the regulation of DNA binding transcription factors (*Nr4a1, Klf2, Nr4a2, Nfil3, Egr3*). At the same time, other notable genes included *Ptgs2*, which regulates prostanoid biosynthesis involved in inflammation and mitogenesis, and *Fosb* and *Jun*, which regulates cell proliferation and differentiation. Moreover, many downregulated are involved in regulating adaptive and innate immune responses, such as *Ccn1, Prdm1 and Midn*. Although the six upregulated DEGs were too few for pathway enrichment analysis, the list of downregulated DEGs were able to be used for GO terms (Fig. 3G), from which the top three pathways included “sprouting angiogenesis”, “response to fluid shear stress”, and “maternal placenta development regulation of transcription”. In the HI samples, both PLS-DA (Fig. 4A) and volcano plots showed robust changes (Fig. 4B). Pathway enrichment analysis using GO terms showed “gland development”, “cardiac myofibril assembly” and “striated muscle cell development” as the top three upregulated pathways (Fig. 4C), while the regulation of endopeptidase activity, negative regulation of hydrolase activity and the regulation of AMPA receptor were amongst the top downregulated pathways (Fig. 4D). Amongst the top upregulated DEGs (Fig. 4E), *Prox1* encodes a member of the homeobox transcription factor family, which has been reported to regulate cell proliferation and circadian rhythm; *Pdgfra*, which encodes a cell surface tyrosine kinase receptor for platelet-derived growth factors, plays a role in cell migration and platelet activation, along with MAPK/ERK signaling pathway activation; *Rreb1*, which encodes a zinc finger transcription factor that binds to RAS-responsive elements and promotes the expression of calcitonin. In the downregulated DEGs (Fig. 4F), genes include *Arc*, which facilitates cell morphogenesis and is considered as a master regulator of synaptic plasticity. In correlation with other DEGs, such as *Fos, Nr4a1, Dusp1, Dusp6*, *Junb*, and *Cst3*, it suggests that cell proliferation and differentiation may be one of the main processes affected by GA/OP. Bar graphs for all up and downregulated DEGs were quantified as TPM transcription levels for quantification (Fig. 4G-H). Overall, both the OB and HI exhibit subtle transcriptomic changes five weeks after GA/OP, with the HI of aged GA/OP mice showing more pronounced alterations compared to sham mice. These findings suggest that GA/OP has long-lasting effects on the brain in aged mice, particularly in vulnerable regions such as hippocampus.

**Figure 8.**
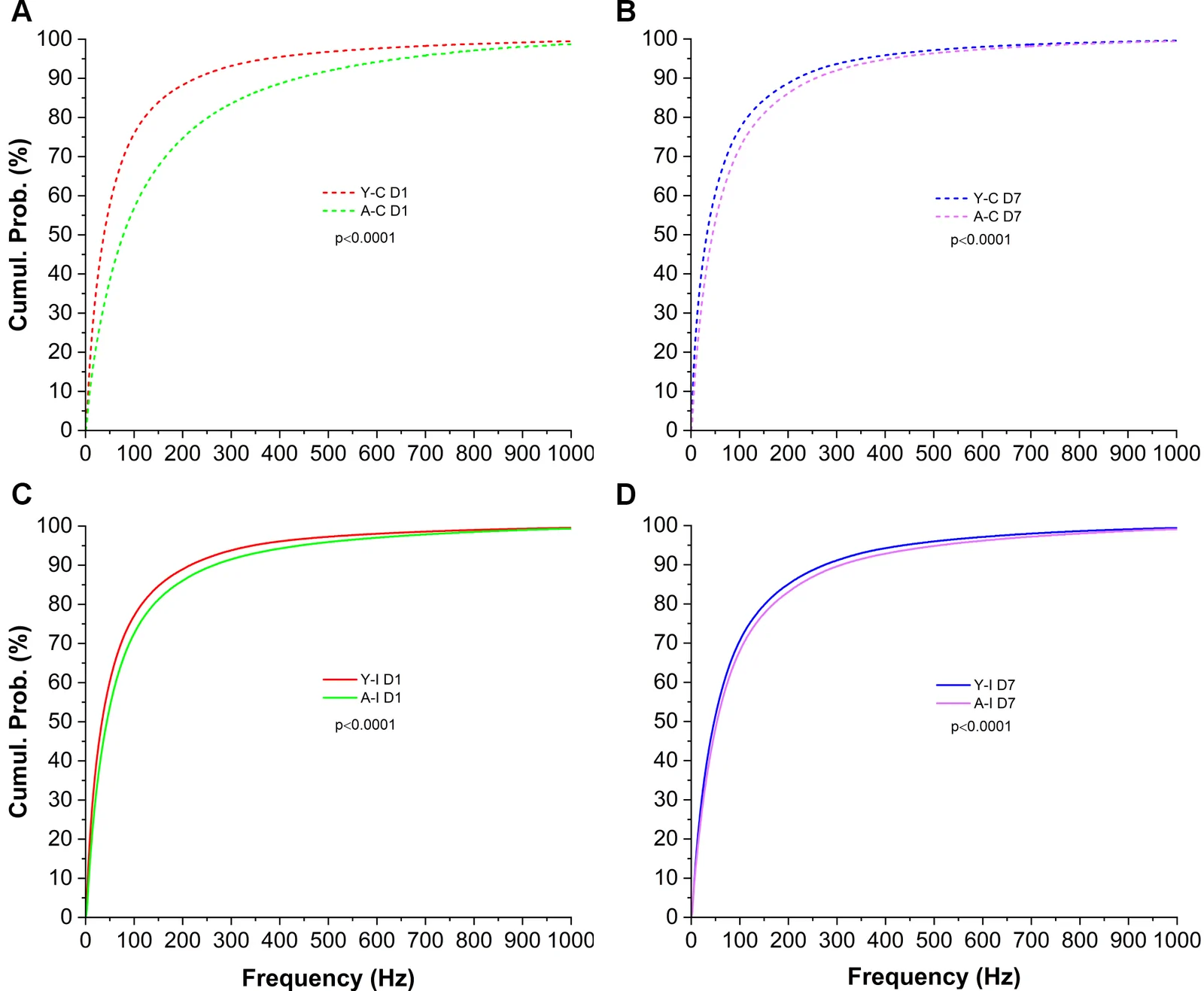
**Effect of age on neuronal excitability in control or isoflurane exposure groups**. (**A-B**) comparison of the cumulative probability of spiking frequency in the control groups of aged vs young mice on day 1 (A) or day 7 (B). (**C-D**) comparison of the cumulative probability of spiking frequency in the isoflurane-treated groups of aged vs young mice on day 1 (C) or day 7 (D) post-isoflurane. Y-I D1: young post-isoflurane day 1 (n=5); Y-C D1: young control day 1 (n=5); Y-I D7: young post-isoflurane day 7 (n=5); Y-C D7: young control day-7 (n=5); A-I D1: aged post-isoflurane day-1 (n=4); A-C D1: aged-control day 1 (n=4); A-I D7: aged post-isoflurane day 7 (n=3); A-C. D7: aged control day 7 (n=4). Comparison between the two groups was analyzed using the Mann-Whitney test.

### Age-dependent changes in molecular pathways are evident 24h after cessation of GA/OP exposure

In clinical settings, age is a key risk factor for POCD and POD following general anesthesia or surgery [47]. Studies in aged mice show that brain vulnerability to GA/OP is age-dependent [48, 49]. In the present study, we compared the transcriptional changes in the OB and HI between aged and young adult mice 24 h after GA/OP cessation. PLS-DA of the normalized transcription counts for OB showed clear separation between aged and young GA/OP groups (Fig. 5A), with various DEGs having an FDR lower than 0.05 (Fig. 5B). Pathway enrichment analysis of the DEGs showed that the top three upregulated pathways were “regulation of membrane potential”, “regulation of monoatomic ion transmembrane transport” and “cellular component assembly” (Fig. 5C). Whereas the top three downregulated pathways were “regulation of neurogenesis”, “positive regulation of cell projection organization” and “actin filament organization” (Fig. 5D). By examining the specific genes featured in the top upregulated heatmap (Fig. 5E), we found that aged mice consistently show higher expression levels of genes involved with the potassium voltage-gated channel (*Kcnh5, Kcnh6, Kcnh7, Kcnb2*), which are involved in neuronal excitability, while *Slc8a1* regulates calcium homeostasis critical for neuronal signaling. In addition, *Ahnak* is a structural protein that modulates TGF-β signaling and calcium dynamics, potentially impacting cellular responses to stress. Moreover, *Wnk3* and *Abcb1a*, which regulate ion transport and blood-brain barrier integrity, respectively, also show increased expression in aged mice. Other notable genes include *C1qb* (complement C1q B chain) and *Ctss* (cathepsin S), suggesting that GA/OP may affect immune complexes and cellular homeostasis. In the downregulated genes (Fig. 5F), *Rgs14* regulates G-protein signaling and plays a role in synaptic plasticity and memory, whereas *Itpka* is involved in calcium signaling and the formation of dendritic spines. Furthermore, *Bdnf* (Brain-derived neurotrophic factor) is an essential neurotrophic factor that supports neuronal survival, synaptic plasticity, and cognitive function, with its increased expression in young mice potentially indicating enhanced neuroprotective mechanisms. *Slit1* participates in axon guidance during neural development, while *Enc1* contributes to cytoskeletal organization and neuronal differentiation. Gene-level expression for each pathway is shown as bar graphs with TPM values on the y-axis (Fig. 5G-H). Taken together, these results demonstrated hyper-activation of the potassium voltage-gated channel in aged OB, along with dysregulation of calcium homeostasis, cell metabolism and decreased synaptic plasticity, highlighting the vulnerability of the OB region to aging effects and GA/OP.

Alongside age-related OB differences, we observed significant HI changes (Fig. 6A-B). Pathway analysis of the HI region showed that regulation of neurogenesis, gliogenesis and small GTPase mediated signal transduction were amongst the top upregulated pathways (Fig. 6C), while RNA splicing, chromatin remodeling and ribonucleoprotein complex biogenesis were amongst the most downregulated pathways (Fig. 6D). In aged GA/OP mice, elevated DEGs including *Gfap, Hapln2, Ldlr, Trpc5,* and *Bhlhe41* are evident (Fig. 6E). *Gfap* (Glial Fibrillary Acidic Protein), a marker of astrocyte activation, is closely tied to neuroinflammation and age-associated brain alterations; *Hapln2*, which facilitates extracellular matrix organization, may drive structural remodeling in the aging olfactory bulb; *Ldlr* (Low-Density Lipoprotein Receptor) participates in lipid metabolism and has been implicated in neurodegenerative pathways, while *Trpc5* encodes a calcium channel critical for neuronal signaling. In addition, the *Bhlhe41* gene was also prominently upregulated, which encodes a transcription factor that influences circadian rhythms and stress responses. Furthermore, both *C1qa* (a complement system component mediating immune responses) and *Hexb* (a lysosomal enzyme), also show higher expression in aged mice, underscoring age-related increases in immune and lysosomal functions. Among the downregulated DEGs (Fig. 6F), notable genes include *Tra2a*, which is involved in RNA splicing regulation, *Malat1*, a long non-coding RNA associated with nuclear organization and gene expression regulation, and *Acin1*, a gene involved in apoptosis and RNA processing. In addition, mitochondrial genes such as *mt-Rnr1* and *mt-Rnr2*, which are critical for mitochondrial ribosomal function and energy production, are also downregulated in the aged GA/OP group. Along with *Mphosph10*, which is associated with ribosome biogenesis, this collection of genes suggests age-related changes in cellular functions such as splicing, apoptosis, ribosomal activity, and mitochondrial dynamics. To enable further quantification, individual genes within each pathway are displayed as bar graphs, with transcript abundance represented in TPM on the y-axis (Supplementary Fig. 8).

Next, we examined the molecular changes of two sham groups in the OB (Supplementary Fig. 9) and HI (Supplementary Fig. 10) regions. In the OB, aging upregulated previously noted potassium channel genes *Kcnh5* and *Kcnh7*. Conversely, aging downregulated genes involved in neurogenesis, including *Islr2* (Immunoglobulin superfamily containing leucine rich repeat 2), *Cd24a*, and *Dynlt1b* (Dynein light chain tctex-type 1). In the HI, aging was associated with upregulation of “fatty acid metabolic process,” “regulation of immune effector process,” and “regulation of membrane potential”. Key DEGs included *Alox5ap* (Arachidonate 5-lipoxygenase activating protein) and *Alox12b* (Arachidonate 12-lipoxygenase, 12R type), involved in arachidonic acid metabolism—linked to inflammatory conditions like asthma, arthritis, and psoriasis. Notable cytochrome P450 (Cyp) family members-related DEGs included *Cyp4f15*, *Cyp2j9*, and *Cyp2d22*, which are essential for drug metabolism and neurotransmitter synthesis [50]. Alternatively, the top downregulated pathways include “mRNA processing”, “chromatin remodeling” and “extracellular matrix organization”. Notable DEGs with the top enrich pathways include *Malat1* (Metastasis associated lung adenocarcinoma transcript 1), *Snrnp70* (Small nuclear ribonucleoprotein U1 subunit 70), *Prpf39* (Pre-mRNA processing factor 39), and *Luc7l3* (Luc7 like 3 pre-mRNA splicing factor). Collectively, these results highlight dysregulated transcriptional activity in aged mice linked to neuroinflammatory signaling, extracellular matrix reorganization, and metabolic processes following GA/OP. Such mechanisms may underline the HI region’s heightened vulnerability to GA/OP in aged individuals.

**Figure 9.**
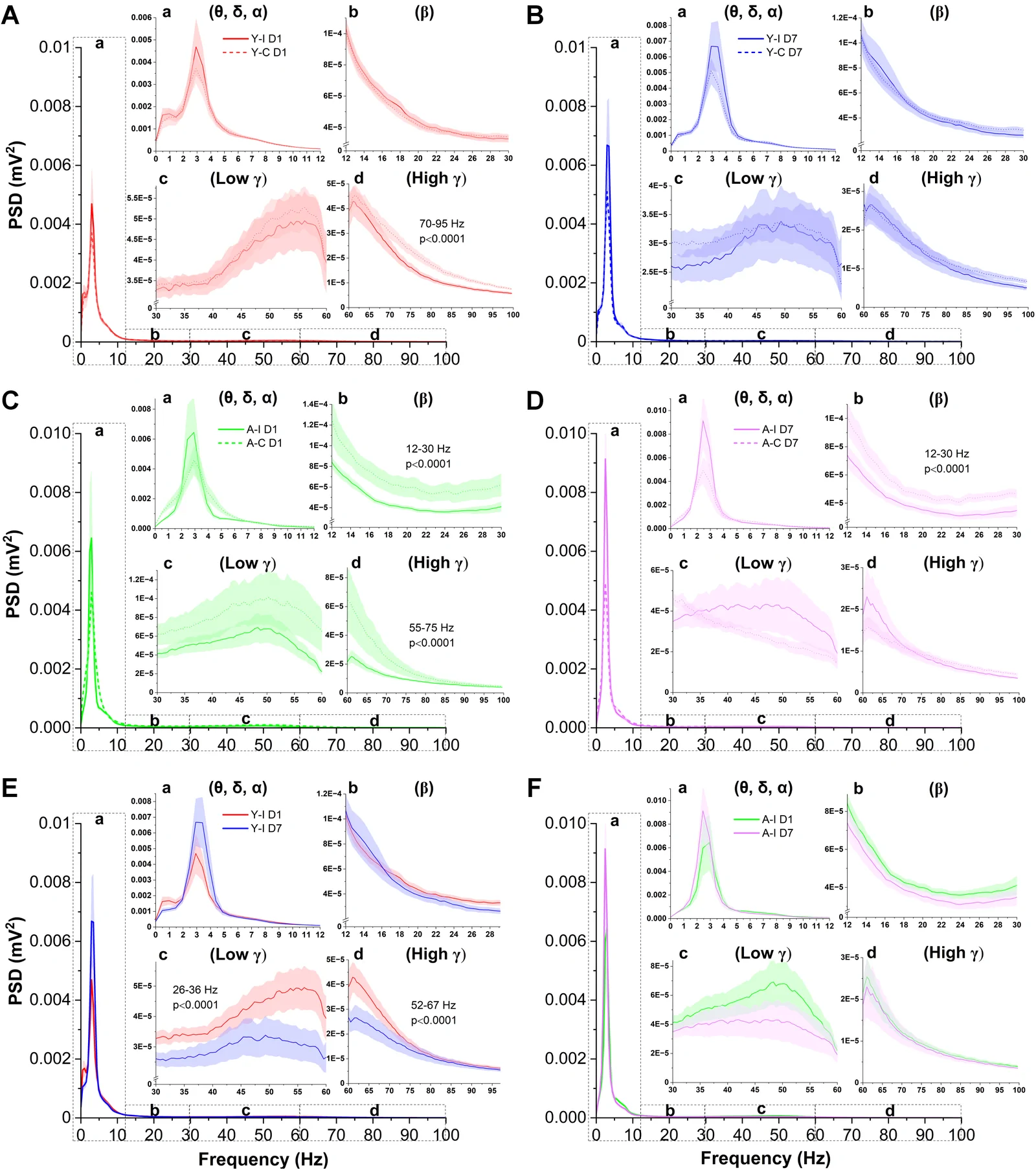
**Acute and chronic effects of isoflurane on neural oscillations of local field potential (LFP) in the OB mitral cell layer**. (**A-D**) Comparison of the power spectrum density (PSD) of LFP oscillatory activities between isoflurane-exposed and control groups on day-1 (A&C) or day-7 (B&D) post-isoflurane in young (A&B) or aged (C&D) animals. Insets are zoom-in from each frequency bands including θ/δ/α (a, 0-12 Hz), β (b, 12-30 Hz), low γ (c, 30-60- Hz), and high γ (d, 60-100 Hz). (**E-F**) Comparison of PSD of LFP oscillatory activities between day 1 and day 7 post-isoflurane in young (E) or aged mice (F). Y-I D1: young post-isoflurane day 1 (n=5); Y-C D1: young control day 1 (n=5); Y-I D7: young post-isoflurane day 7 (n=5); Y-C D7: young control day-7 (n=5); A-I D1: aged post-isoflurane day-1 (n=4); A-C D1: aged-control day 1 (n=4); A-I D7: aged post-isoflurane day 7 (n=3); A-C D7: aged control day 7 (n=4). PSD: Power Spectral Density. Values are presented as mean±SE. Comparison between the two groups was analyzed using the Mann-Whitney test.

**Figure 10.**
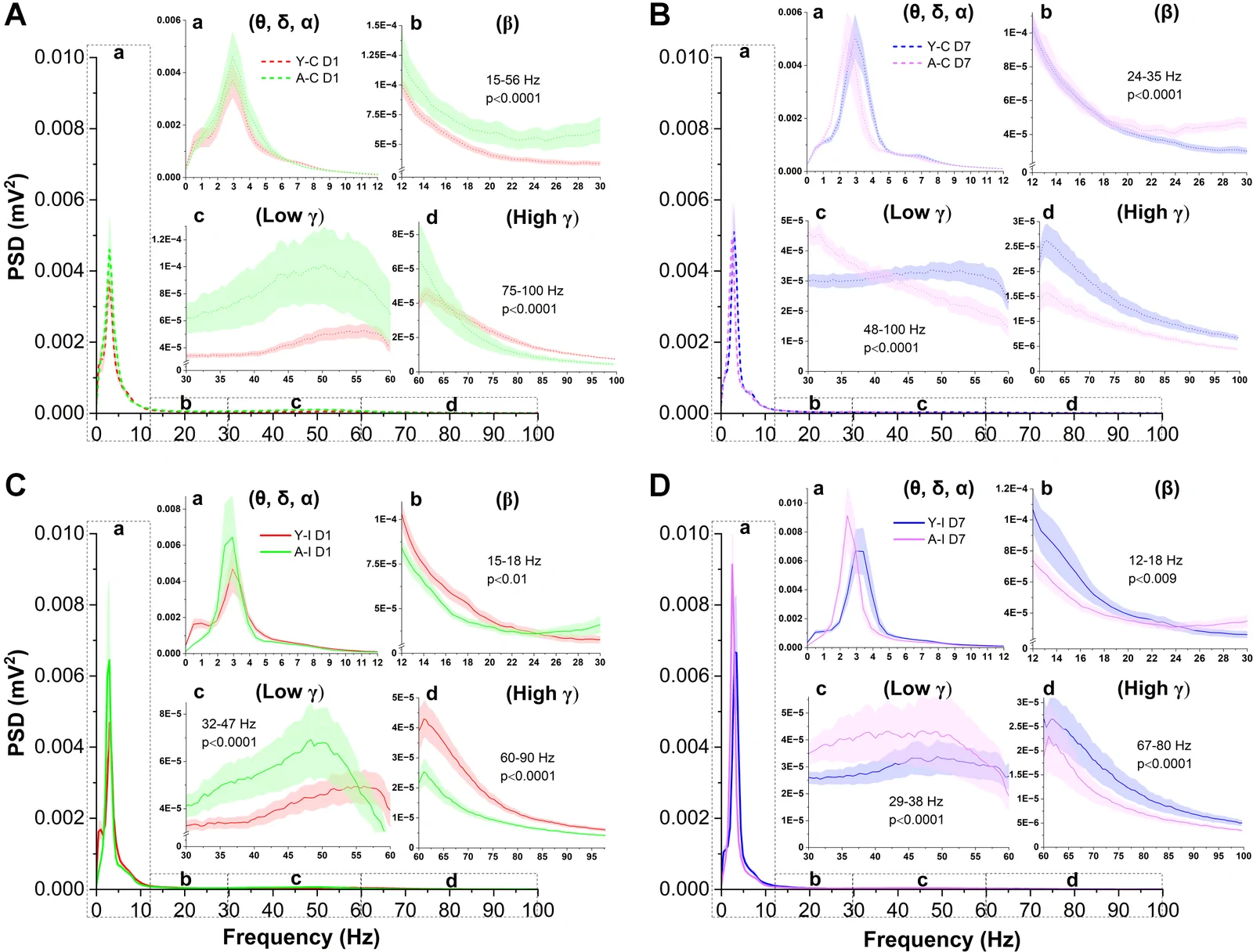
**Effect of age on oscillatory activities of LFP in control or isoflurane exposure groups**. (**A-B**) comparison of the PSD of LFP oscillatory activities in the control groups of aged vs young mice on day 1 (A) or day 7 (B). (**C-D**) comparison of the PSD of LFP oscillatory activities in the isoflurane-treated groups of aged vs young mice on day 1 (C) or day 7 (D) post-isoflurane. Insets are zoom-in from each frequency bands including θ/δ/α (a, 0-12 Hz), β (b, 12-30 Hz), low γ (c, 30-60- Hz), and high γ (d, 60-100 Hz). Y-I D1: young post-isoflurane day 1 (n=5); Y-C D1: young control day 1 (n=5); Y-I D7: young post-isoflurane day 7 (n=5); Y-C D7: young control day-7 (n=5); A-I D1: aged post-isoflurane day-1 (n=4); A-C D1: aged-control day 1 (n=4); A-I D7: aged post-isoflurane day 7 (n=3); A-C. D7: aged control day 7 (N=4). PSD: Power Spectral Density. Values are presented as mean±SE. Comparison between the two groups was analyzed using the Mann-Whitney test.

### Anesthesia impairs age-dependent neuronal excitability in the OB of awake mice

To assess acute and chronic effects of general anesthesia with isoflurane on neuronal excitability and network operations in the OB, we performed *in vivo* electrophysiological recordings with a neural probe of linearly arranged 32 channels targeting two time points after 2-hour GA exposure (day 1 and day 7) in awake but head-fixed animals (Supplementary Fig. 11A). Spontaneous spiking activities and LFP oscillatory power at different frequency bands from 32 channels were recorded and analyzed to assess neuronal excitability and network operation (Supplementary Fig. 11B).

In young (Y) adult animals, 2-hour isoflurane (I, n=4 mice) exposure left-shifted the accumulative probability of spiking frequency compared to the control (C) group (n=5 mice) on day 1 post-isoflurane, suggesting a reduced neuronal excitability in the MCL (P<0.008, Y-I vs Y-C, n=14 recordings in control and n=14 in isoflurane group) (Fig. 7A red solid line vs red dotted line). However, the isoflurane effect was reversed on day 7 post-isoflurane as the accumulative probability of neuronal spiking frequency for the Y-I group was right-shifted compared to the control group (P<0.0001 n=14 recordings in control and n=14 in isoflurane group) (Fig. 7B blue solid line vs blue dotted line), indicating neuronal hyperexcitation. Similar results were observed in the aged animals (P<0.0001 day 7, n=7 recordings in control and n=11 in isoflurane group) (Fig. 7C-D). Surprisingly, the isoflurane effects on day 1 were much more profound in the aged animals compared to the young adults. This may be due to the hyperexcitability of OB neurons in the aged control mice compared to the young control group (P<0.0001, A-I vs A-C, n=8 recordings in control and n=12 in isoflurane group) (Fig. 7C, inset). During the course following isoflurane treatment, the OB mitral cell spontaneous spiking activities increased from day 1 to day 7 in both young and aged groups (Fig. 7E-F), indicating a chronic enhancement of neuronal excitability following 2-hour isoflurane anesthesia.

To assess the potential age-dependence of these isoflurane effects, data from the aged animal groups were compared to young mice on post-isoflurane day 1 or day 7. Collectively, aged mice showed higher average spontaneous spike frequencies compared to young animals on both day 1 and day 7 in both control (Fig. 8A-B) and isoflurane-exposed (Fig. 8C-D) conditions, suggesting higher excitability of OB MCL cells in the aged mice. But this age-dependent difference on day 1 was more profound compared to day 7 in the control condition.

### Anesthesia exposure leads to age-dependent disruption of the OB neuronal network

Since GABA_A_ receptors as chloride-permeable ligand-gated ion channels not only contribute to the majority of inhibition in the brain including the OB but have also been identified as the crucial action target of general anesthetics including isoflurane [51], OB network operation is potentially subject to long-term alterations by isoflurane. To test this, we analyzed the oscillatory activities of LFP recorded by our 32-channel neural probes as these rhythmic events at different frequency bands represent network synchronization and interactions between excitatory and inhibitory neurons but with distinct circuit origins [46, 52].

In the young mice as shown in Figure 9A, the oscillation strength as measured by the power spectrum density of LFP in the high-gamma frequency range of 70-95 Hz on post-isoflurane day 1 was significantly less compared to the control group (p<0.0001, n=14) while no difference was detected between these two animal groups on post-isoflurane day 7 (Fig. 9B). By contrast, the oscillations in the beta frequency range of 12-30 Hz in aged mice on both post-isoflurane day 1 and day 7 were significantly weaker compared to the control group (p<0.0001, n=12, 11, and 8, respectively) (Fig. 9C-D). Furthermore, similar alterations in the high gamma frequency range of 55-75 Hz were observed in aged mice on both post-isoflurane day 1 compared to the control group (p<0.0001, n=12 and 8 respectively) (Fig. 9C-D). Surprisingly, the isoflurane effects on oscillatory activities in the gamma frequency range 26-36 Hz and 52-67 Hz in young mice developed over time because the strength of these events in these frequency ranges reduced in the isoflurane-exposed group on day 7 compared to that on post-isoflurane day 1 (p<0.0001, n=14recordings) (Fig. 9E). These effects were not observed in the aged animal group (Fig. 9F). These results suggest a particular vulnerability of OB network operation to isoflurane lasting effects in aged mice. This is consistent with the direct comparison of the oscillatory activities between aged and young mice on post-isoflurane day 1 or day 7 (Fig. 10A-B), respectively. Specifically, oscillatory activity strength in the beta frequency (15-18 Hz) and gamma frequency (60-90 Hz) was significantly less in age mice on post-isoflurane day 1 (Fig. 10A) where similar results in a slightly different frequency ranges were revealed for the post-isoflurane day 7 data comparison (Fig. 10B).

## DISCUSSION

This study examined the lasting effects of isoflurane exposure and abdominal surgery on olfactory and cognitive function in aged (20-month-old) C57BL/6 mice. Results showed significant and persistent isoflurane/OP-induced olfactory impairment, as indicated by deficits in both odor recall (OM test) and detection sensitivity (BF test). Notably, isoflurane alone also caused delayed but significant impairment, especially in higher-order processing assessed by the OM test. While partial recovery in odor sensitivity was observed in the BF test during later stages, OM deficits persisted, suggesting long-term disruption. These findings point to isoflurane-induced OD, potentially mediated by neuroinflammation and blood-brain barrier disruption, as reported in prior studies [53-55]. The combined impact of surgical stress and anesthesia may further exacerbate OD. The delayed onset of symptoms with GA alone also raises the possibility of an anesthetic-triggered neurodegenerative process, aligning with earlier reports of persistent memory impairment in rodents [56-58]. Our findings partially align with those of Lan et al., who reported significantly lower olfactory recognition thresholds and memory impairment seven days after surgery in elderly patients [4]. Notably, their study used an intravenous anesthetic cocktail, not isoflurane, suggesting that our observations may extend to other anesthetics. Our longer study timeline also offers insight into the potential long-term effects of GA/OP and associated molecular changes. However, anesthetics may differ in their impact: a comparative study in elderly patients undergoing laparoscopic cholecystectomy revealed varying POCD incidence and plasma cytokine levels across isoflurane, propofol, and sevoflurane, implying distinct mechanisms [59]. Similarly, Zuo et al. showed that neonatal rats exposed to isoflurane or sevoflurane exhibited impaired Morris water maze performance and reduced neurogenesis [60]. Another study in aged rats found cognitive deficits post-abdominal surgery and sevoflurane exposure, even after brief anesthesia [61]. Together, these studies suggest both shared and unique pathways of anesthetic-induced neurotoxicity, highlighting the need for further molecular investigation.

Our findings in young adult mice suggest that neurological deficits induced by short-term GA/OP are age dependent. This contrasts with Liufu et al., who reported increased latency in the buried food test in 9-month-old female mice 9 hours after GA/OP—possibly due to the earlier timepoint, sex differences, or older age of their cohort [20]. Similarly, Zhang et al. observed reduced sniffing time in the block test following laparotomy and isoflurane exposure in 4-month-old female mice, indicating olfactory dysfunction [6], though sex and age differences may again explain the discrepancy. Regarding cognitive function, our results are consistent with studies showing no impairment in Barnes maze performance up to 42 days after 2h of 1.4% isoflurane exposure in young adult male mice [62]. Borgstedt et al. also found no behavioral changes in 10-month-old mice after similar exposure [63]. Overall, the limited studies on OD after GA/OP vary in methodology, age, and sex, which likely accounts for differences in outcomes.

The assessment of neuromuscular and locomotor functions revealed a complex picture of GA/OP's effects in aged mice. Twenty-four hours cessation following GA/OP, the aged mice exhibited increased frailty, as indicated by significantly reduced grip strength indicating an impact on neuromuscular function. Interestingly, GA exposure alone did not initially affect grip strength, but some mice showed poorer performance in the Rotarod test, highlighting a potential vulnerability to GA. Over time, the GA/OP mice continued to display impaired neuromuscular function and locomotor ability, further supporting the notion of prolonged frailty after surgical intervention. While mice exposed to isoflurane alone initially showed no locomotor deficits, they exhibited a transient impairment at 12 days before recovering. Despite these impairments in grip strength and Rotarod performance in the GA/OP group, spontaneous activity remained unaffected in both the GA/OP and GA exposure groups, suggesting that GA with isoflurane and surgery have a selective impact on specific aspects of motor function rather than causing a general reduction in activity.

The examination of cognitive functions via the Nest building test, Y-maze, and Novel object recognition test revealed a delayed cognitive impairment in aged mice following GA/OP. While no significant differences were observed during the first week after surgery, the GA/OP mice displayed impaired nest building abilities at 11 days, with some mice exhibiting progressively worsening apathy-like symptoms by day 34. The NB task is a simple rodent test for self-care [34, 35], in which a low score for the nest quality built by an animal indicates apathy-like behavior. Apathy is the most common behavioral and psychological symptom in AD and other neurodegenerative diseases, including frontotemporal dementia and PD. In patients, apathy can include loss of motivation, initiative, and interest, listlessness and indifference, flattening of emotions, and absence of drive and passion. Researchers have later refined this to a reduction in goal-directed behaviors. In animals, specific symptoms of apathy-like behavior have been modelled, including goal-directed or nest-building behavior, which are seen as indicative of proxies for motivation and daily activities. Reduced NB behavior has been reported for various transgenic mouse models of AD [64-66]. Similarly, Y-maze and NOR testing also revealed impaired spatial and non-spatial memory in week 5 post-GA/OP. These findings, combined with the previous observations of long-lasting OD and increased frailty, indicate that GA and post-operation cause a delayed onset of cognitive impairment in aged mice. However, a few research studies have shown that GA does not result in long-term cognitive deficits in aged mice [67, 68]. Our differing conclusions may be due to variations in surgery and isoflurane concentration. Moreover, the delayed effect that we observed in the present study highlights the importance of when assessments take place, which also contributes to the conflicting conclusions in prior studies.

The RNAseq analysis of the OB following GA/OP in aged mice revealed substantial transcriptional changes indicative of cellular stress and altered molecular processes. PLS-DA demonstrated a clear separation between GA/OP and Sham groups, confirming a distinct gene expression profile shift. The upregulation of pathways related to protein degradation, mRNA processing, and neurogenesis regulation suggests an adaptive response to surgical stress, potentially mitigating damage or facilitating recovery. Conversely, the downregulation of pathways associated with ribonucleo-protein complex biogenesis and RNA processing highlights a disruption in essential cellular functions. Notably, key DEGs such as *Wnt10b, Snhg15, Prkn,* and *Ddit3* point toward mechanisms involving Wnt signaling, non-coding RNA activity, proteostasis, and stress response, all of which have implications for neurodegenerative processes. The observed dysregulation in RNA splicing and protein modification—evidenced by genes such as *Cirbp, Rbm11*, and *Spsb1*—suggests that altered post-transcriptional and post-translational regulation may contribute to OB dysfunction. Additionally, downregulated DEGs involved in mRNA binding and cell cycle regulation further emphasize the broad molecular disturbances induced by GA/OP. These findings underscore the vulnerability of the OB to post-GA/OP, potentially linking impaired olfactory function to broader neurodegenerative and metabolic pathways. In the HI region, transcription changes rapidly on the first day after cessation of GA/OP, but functional effects emerge only weeks later. The reason molecular changes usually precede behavioral changes in biological systems - especially in the context of diseases like neurodegeneration, psychiatric disorders, or injury [69-71] - is due to the hierarchical nature of biological responses. Thus, molecular changes are early indicators of stress or pathology, while behavioral changes are late-stage manifestations of accumulated dysfunction. Understanding this progression is critical for early diagnosis and intervention.

Five weeks after GA/OP cessation, transcriptomic analysis revealed persistent molecular changes in the OB and HI, indicating long-term impacts. In the OB, PLS-DA showed clear group separation, but few DEGs were detected—mostly downregulated and enriched in pathways related to angiogenesis, shear stress, and transcriptional regulation. Upregulated DEGs were too few for enrichment analysis but linked to microtubule motor activity, DNA damage response, and translation. In contrast, the HI showed more pronounced changes, with upregulated pathways involving gland development and muscle differentiation, and downregulated ones tied to proteolysis and AMPA receptor activity. Key upregulated genes (*Prox1*, *Pdgfra*, *Rreb1*) implicated cell proliferation and signaling, while downregulated genes (Arc, Fos, Nr4a1) suggested impaired synaptic plasticity. The distinct DEG profiles underscore the HI’s heightened vulnerability to GA/OP-induced stress, suggesting these long-term effects may contribute to cognitive and neurodevelopmental deficits in aging.

Transcriptional profiling of the OB and HI from aged (18-month-old) and young (3-month-old) mice following 2h GA/OP revealed marked age-related molecular differences. Aged mice showed heightened vulnerability to GA/OP stress, with the OB displaying distinct upregulation of immune-related pathways, including responses to biotic stimuli and viral infections—indicative of increased neuroinflammatory signaling. Genes associated with potassium voltage-gated channels and calcium homeostasis were also upregulated, suggesting enhanced neuronal excitability and potential synaptic dysfunction, both relevant to AD pathology [72, 73]. Conversely, pathways related to neurogenesis and cytoskeletal organization were downregulated, with reduced expression of plasticity-associated genes, implying impaired regenerative capacity and olfactory decline. Similar trends were observed in HI, where aged mice exhibited broad transcriptional shifts, including upregulation of astrocyte and immune genes, pointing to neuroinflammation and extracellular matrix remodeling. Downregulated pathways involved RNA splicing, chromatin remodeling, and mitochondrial function, significantly reduced, indicating disrupted homeostasis and energy metabolism. Together, these findings highlight the pronounced susceptibility of aged brains to GA/OP, marked by immune activation, synaptic instability, and metabolic dysfunction in regions critical for cognition and sensory processing. They underscore the need for age-tailored strategies to mitigate postoperative cognitive and olfactory impairments.

At the physiological level, we observed age- and timing-dependent isoflurane effects on neuronal excitability and network operations in the OB. At 24 hours post-GA, the neuronal excitability in the OB mitral cell layers of aged animals was robustly suppressed compared to age-matched sham group or corresponding isoflurane-treated young mice. This is consistent with our observation of upregulated expression levels of genes involved with the potassium voltage-gated channel (*Kcnh5, Kcnh6, Kcnh7*) in aged mice since these ion channels negatively control neuronal excitability [74]. Intriguingly, these effects were reversed on day 7 post-GA, i.e., neuronal excitability was upregulated in both young and aged mice compared to corresponding sham groups, correlating with our findings from odor-based behavioral tests and aligning with a recent report of hyperexcitability of OB mitral cells in young adult mice on day 2 post-repetitive- isoflurane-exposures [75]. Although what causes this hypo- to hyperexcitability switch from post-GA day 1 to day 7 is unknown, the downregulation of gene encoding AMPA receptors as we observed at 5 weeks post-GA/OP may play a contributive role because downregulated AMPA receptor activities lead to reduced inhibitory feedback from OB inhibitory GABAergic interneurons, most of which are driven by the activation of AMPA receptors in response to glutamate released from excitatory projection neurons or olfactory sensory neurons[26].

Given the crucial role of mitral cells in the operation of complex neural circuits formed among excitatory and inhibitory neurons in the OB [26, 76], the lasting impact of GA/OP on their excitability conceivably lead to alterations of local network operations. Our analysis of oscillatory activities of LFP supports this prediction. Since isoflurane may not affect only mitral cells [75], the age- and timing-dependence of its effects do not align with alterations in mitral cell excitability. Although the lasting impact of isoflurane on other OB cell types warrants future research endeavor, it is sensible to speculate deduce the contributive role of OB network dysfunction in the GA/OP-induced OD. Taken together, our physiological data provided the first experimental evidence supporting the lasting and age-dependent detrimental effects of GA/OP on neuronal excitability and network operation in the OB and bridged the relevant molecular and behavioral level findings.

One limitation of our study is the lack of analysis by biological sex, a known factor in neurological outcomes following GA/OP. However, evidence on which sex is more vulnerable remains conflicting. A meta-analysis found that male cardiac surgery patients were more likely to develop postoperative delirium (POD) within the first 3 days than age-matched females [77], consistent with earlier findings in hip fracture patients [78]. In contrast, a more recent pooled analysis reported that females were at least 1.54 times more likely to develop POD [79]. Another study found older male APOE4 carriers were more susceptible to postoperative cognitive dysfunction (POCD) than female carriers [80]. These discrepancies may reflect differences in timing, age, or surgery type. Similarly, animal studies yield mixed results: female 5xFAD mice showed cognitive deficits after sevoflurane and surgery, whereas males did not [81]; conversely, male Sprague-Dawley rats exhibited more severe long-term impairments after isoflurane exposure [82]. Together, these findings highlight species- and context-dependent sex differences, warranting further investigation.

In conclusion, our study demonstrated that mice undergoing abdominal surgery with 2h of isoflurane exposure developed long-lasting and age-dependent olfactory dysfunction, increased frailty, and delayed onset of cognitive impairment compared to sham controls along with underlying transcriptomic, neuronal and network alterations. These findings highlight the clinical relevance of postoperative olfactory and cognitive deficits in elderly patients and underscore the need for further research to identify potential therapeutic strategies aimed at mitigating these neurological complications and improving the outcomes in vulnerable populations.

## Supplementary Materials

The Supplementary data can be found online at: www.aginganddisease.org/EN/10.14336/AD.2025.0596.

## Data Availability

All data needed to evaluate the conclusions in the paper are present in the paper and/or the Supplementary Materials. All RNA-seq data were deposited in the Gene Expression Omnibus (GEO) under the accession number: GSE 297195 at www.ncbi.nlm.nih.gov/geo/query/acc.cgi?acc=GSE297195.
