# Supplementary material for: Age-dependent Transcriptional and Circuit Alterations in the brain Underlie Post-Anesthesia Neurobehavioral Dysfunction
Source: Aging Dis. 2025 Jun 6;17(4):2131–53. doi: 10.14336/AD.2025.0596 (PMC13256344; doi:10.14336/AD.2025.0596)
Supplement: Supplementary file 1 — The Supplementary data can be found online at: www.aginganddisease.org/EN/10.14336/AD.2025.0596. [file AD-17-4-2131-s.pdf]

SUPPLEMENTARY DATA

Age-dependent Transcriptional and Circuit Alterations in the brain Underlie Post-Anesthesia Neurobehavioral Dysfunction

Yun Li, Cosar Uzun, Syed Taufiqul Islam, Balaji Krishnamachary, Hangnoh Lee, Zihui Wang, Hui Li, Shaolin Liu, Junfang Wu

© 2025. Li Y et al. Published online at http://www.aginganddisease.org/EN/10.14336/AD.2025.0596

SUPPLEMENTARY DATA

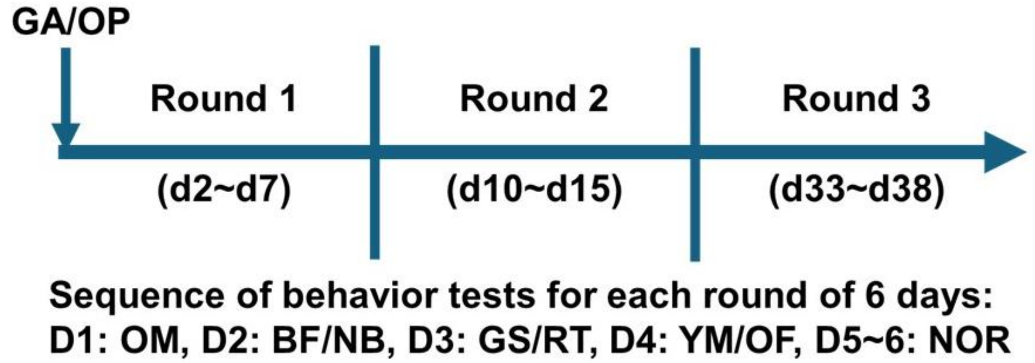

Supplementary Figure 1. **Schematic illustration of the experimental workflow for a battery of neurological behavioral tests**. OM: odor memory, BF: buried food, NB: nest building, GS: grip strength, RT: rotarod, YM: Y maze, OF: open field, NOR: novel object recognition.

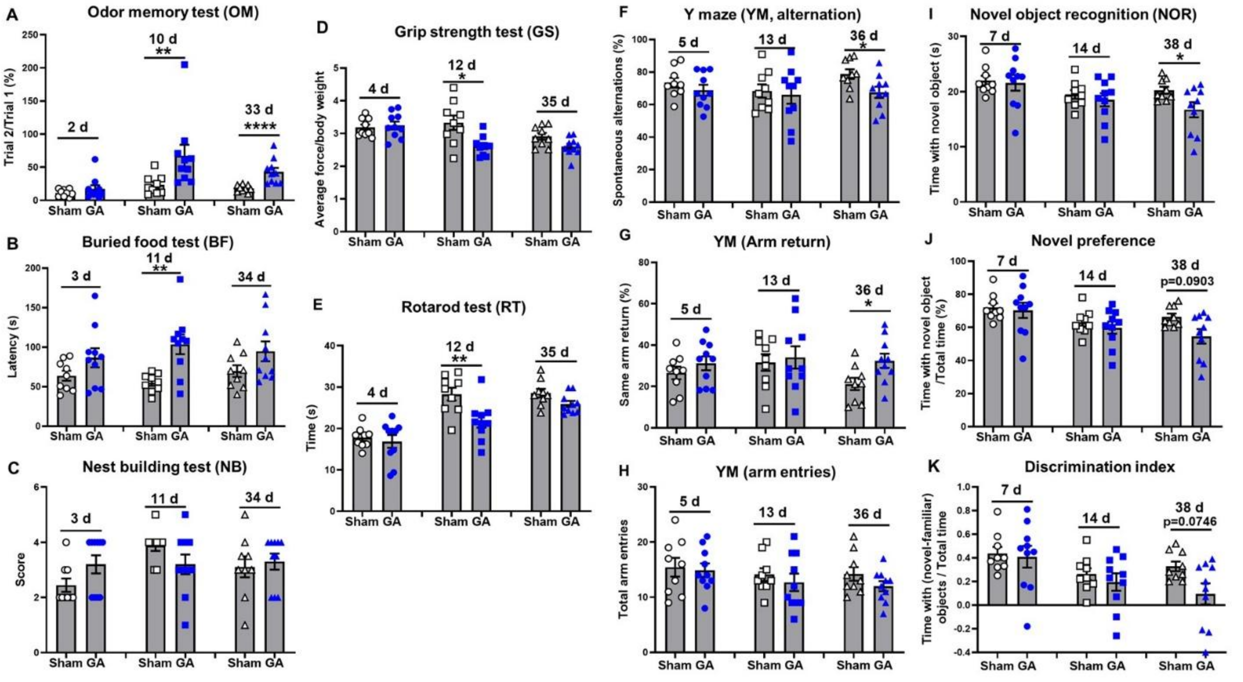

Supplementary Figure 2. General anesthesia (GA) triggers transient neurological dysfunction in aged mice. Aged (18–20-month-old) C57BL/6 male mice were subjected to 2 hours isoflurane exposure without surgical procedure. **(A-B)** Delayed impairment of olfactory function was observed in GA mice, as signified by disruption to odor memory (OM) test (A) and increased latency to find pellets in buried food (BF) test (B). **(C)** Animal general well-being was examined with the nest building (NB) test, which was not significantly affected by GA. **(D-E)** Neuromuscular function showed temporary deficits in grip strength (GS) test (D) and Rotarod (RT) test (E) at 12d after GA cessation. **(F-H)** The spatial memory of aged mice was assessed using the Y-maze (YM) test, which revealed a delayed detrimental effect on spontaneous alternations (F) and arm return behavior (G) at 36 days, while the total number of arm entries remained unchanged (H). **(I-K)** Novel object recognition (NOR) testing revealed delayed GA-induced deficits in non-spatial memory, as evidenced by reduced time spent exploring the novel object (I), as well as decreased novel object preference (J) and discrimination index (K). n=9-10/group. * $p < 0.05$, ** $p < 0.01$, **** $p < 0.0001$. Data was analyzed with Mann Whitney U-test for nonparametric data in experiments (A,C,D,F,G,J,K) or unpaired t-test for (B,E,H,I).

© 2025. Li Y et al. Published online at http://www.aginganddisease.org/EN/10.14336/AD.2025.0596

SUPPLEMENTARY DATA

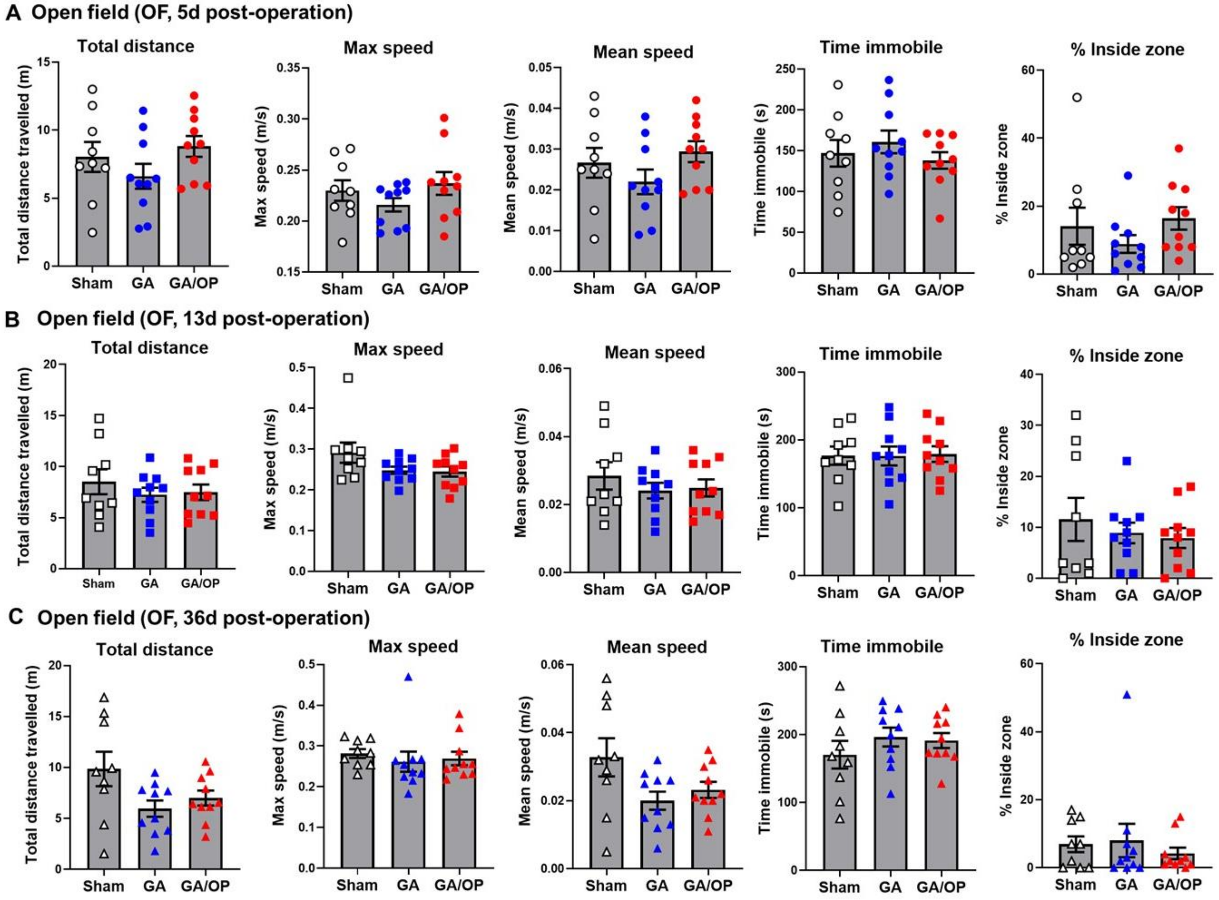

Supplementary Figure 3. **Short-term general anesthesia, with or without surgery, does not affect the spontaneous locomotor activity of aged mice.** The AnyMaze behavior system was used to record and analyze spontaneous activity in an open field apparatus at 5 (A), 13 (B), and 36 days (C) after GA cessation. n=9-10 mice/group.

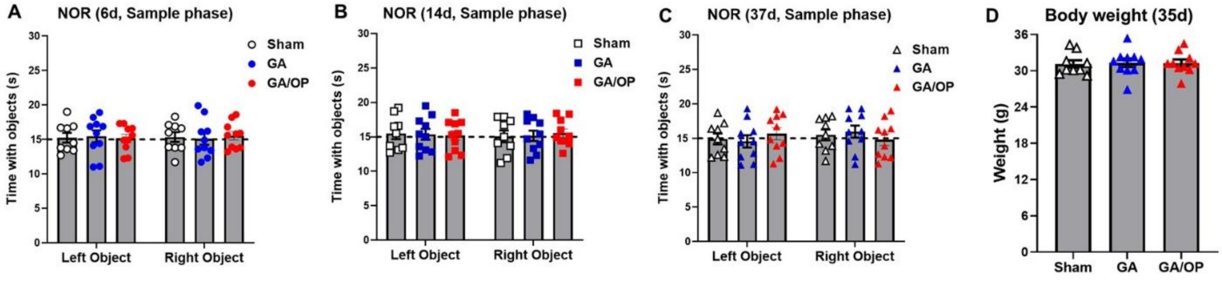

Supplementary Figure 4. **There were no differences between the groups in the sample phase of the novel object recognition (NOR) test or in final body weight. (A-C)** General anesthesia, with or without surgery (GA/OP, GA) did not affect placement preference when exploring the familiar objects at day 6 (A), 14 (B) and 37 (C) after GA cessation. **(D)** GA and GA/OP had no effects on the body weight of aged mice at 35 d after GA cessation. n=9-10 mice/group.

© 2025. Li Y et al. Published online at http://www.aginganddisease.org/EN/10.14336/AD.2025.0596

SUPPLEMENTARY DATA

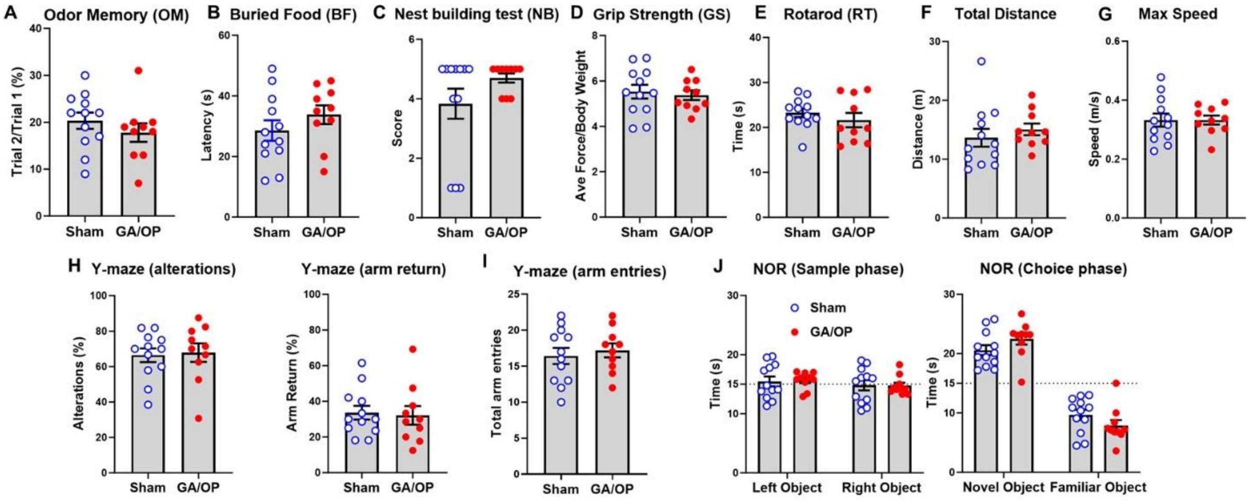

Supplementary Figure 5. **Supplementary Figure 5. Short-term general anesthesia/surgical operation (GA/OP) has no effect on the neurological functions of young adult mice.** Young adult (6–9-week-old) C57BL/6 male mice underwent laparotomy followed by 2 hours of isoflurane exposure and were assessed for a battery of neurological functions 24 hours after cessation of GA/OP. **(A-B)** Olfactory function remained intact in mice during the 2-3 days post-GA/OP, as demonstrated by the odor memory (OM, A) and the buried food (BF) tests (B). **(C)** General well-being was assessed on day 3 using the nest building (NB) test, which showed no impairment following GA/OP. **(D-E)** Neuromuscular function showed no deficits in the grip strength (GS) test (D) or the Rotarod (RT) test (E) at 4 days post GA/OP. **(F-G)** Total distance traveled and maximum speed in the open field test showed no differences between GA/OP mice and sham controls at day 5 post-procedure. **(H-I)** Spatial memory was assessed using the Y-maze (YM) test, which revealed no differences in spontaneous alternations or arm returns (H), and the total number of arm entries remained unchanged (I). **(J)** Novel object recognition (NOR) testing revealed no changes in non-spatial memory, as shown by similar times spent exploring the novel object during the choice phase. n=12 (Sham) and 10 (GA/OP) mice. Data was analyzed with Mann Whitney U-test for nonparametric data in experiments (A,C,D,H) or unpaired t-test for (B,E,F,G,I). Two-way ANOVA followed by Tukey's post-hoc comparison was used for analysis of NOR data (J).

© 2025. Li Y et al. Published online at http://www.aginganddisease.org/EN/10.14336/AD.2025.0596

SUPPLEMENTARY DATA

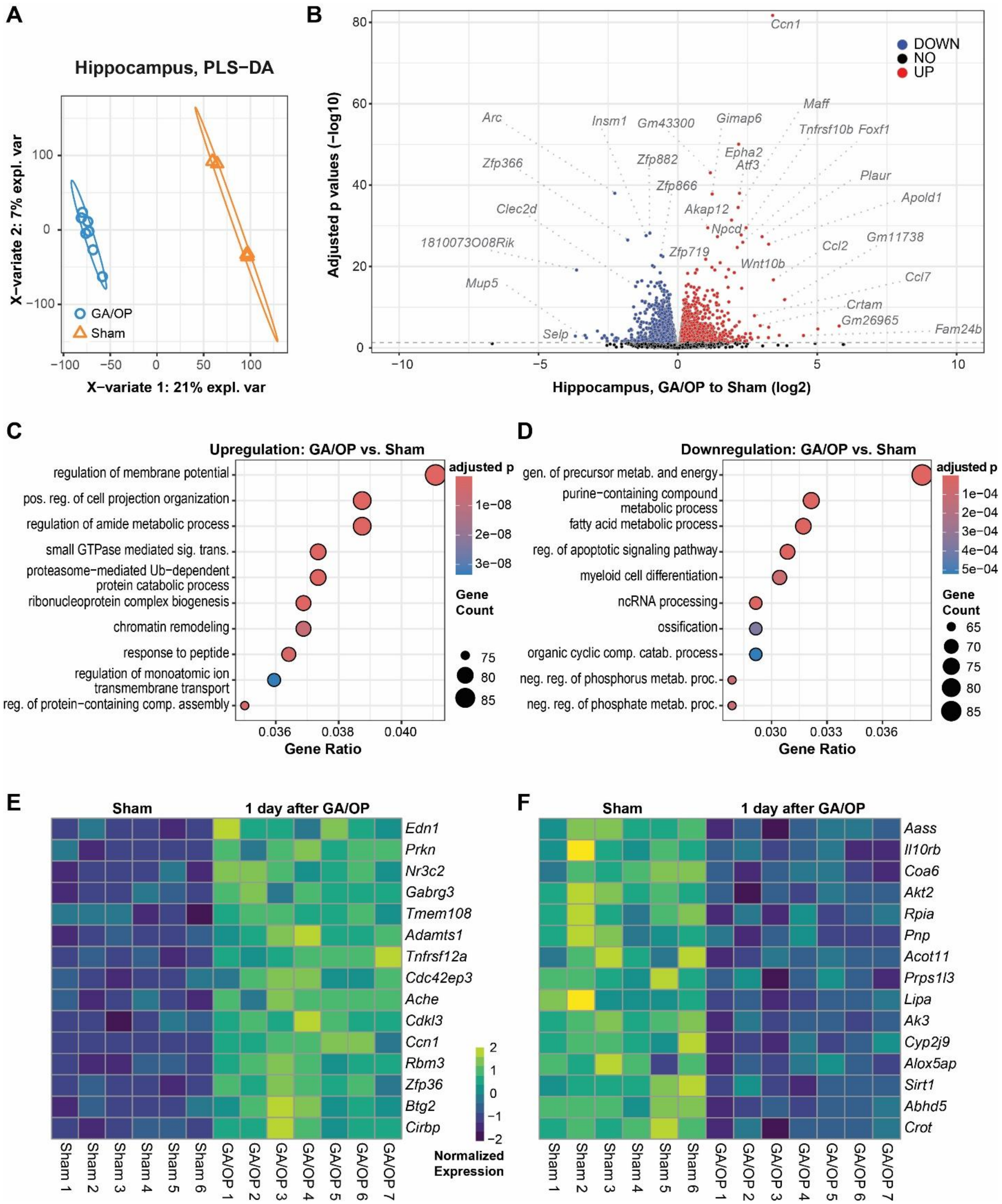

Supplementary Figure 6. Short-term general anesthesia/surgical operation (GA/OP) in aged mice induces molecular changes in the hippocampus (HI) 24h after cessation. (A) PLS-DA plot for normalized transcriptome genes shows sample clustering by groups. **(B)** Volcano plot of all genes after pairwise comparison of Aged GA/OP vs. Aged Sham. **(C-D)** GO terms pathway enrichment analysis of downregulated DEGs **(E-F)** Heatmap of DEGs involved with the top 3 up- and down-regulated genes. n=7 (GA/OP) and 6 (Sham) mice.

© 2025. Li Y et al. Published online at http://www.aginganddisease.org/EN/10.14336/AD.2025.0596

SUPPLEMENTARY DATA

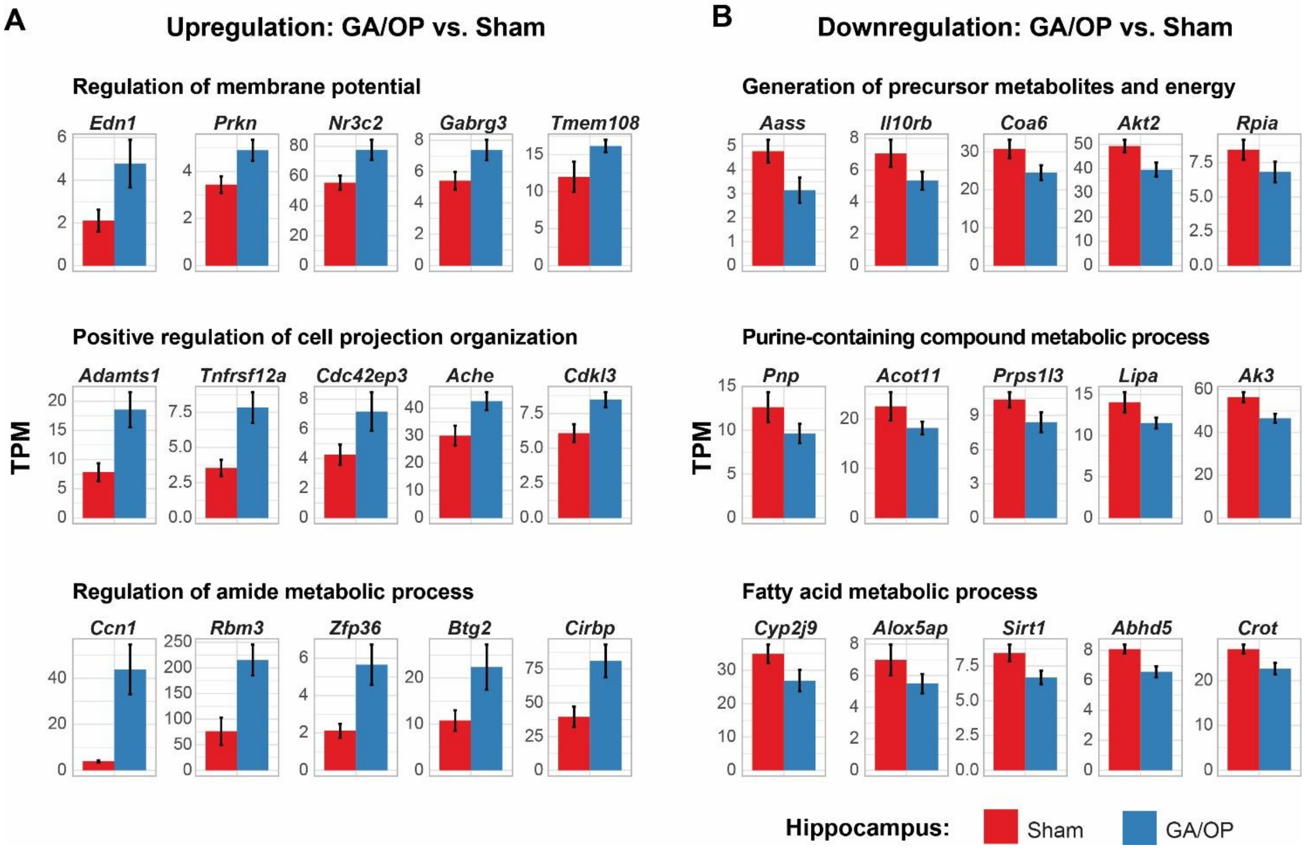

Supplementary Figure 7. Expression levels of top regulated DEGs in the hippocampus (HI) of aged mice at 24h after GA/OP. (A) Upregulation pathways. **(B)** Downregulation pathways. Bar graph of genes after quantification by TPM. n=7 (GA/OP) and 6 (Sham) mice.

© 2025. Li Y et al. Published online at http://www.aginganddisease.org/EN/10.14336/AD.2025.0596

SUPPLEMENTARY DATA

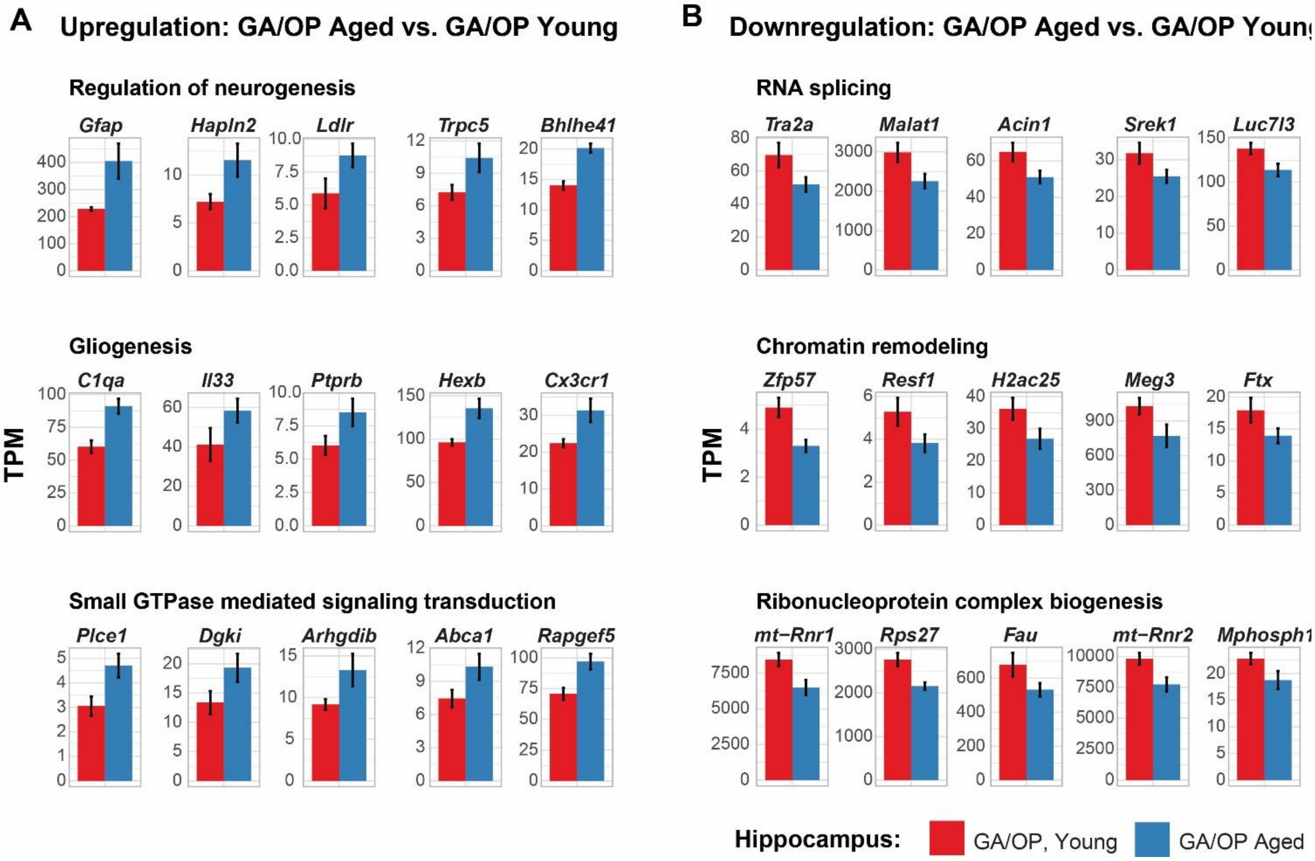

Supplementary Figure 8. Expression levels of top age-dependent DEGs in the hippocampus (HI) region at 24h after GA/OP. (A) Upregulation pathways. **(B)** Downregulation pathways. n=5 (Young GA/OP) and 7 (Aged GA/OP) mice.

© 2025. Li Y et al. Published online at http://www.aginganddisease.org/EN/10.14336/AD.2025.0596

SUPPLEMENTARY DATA

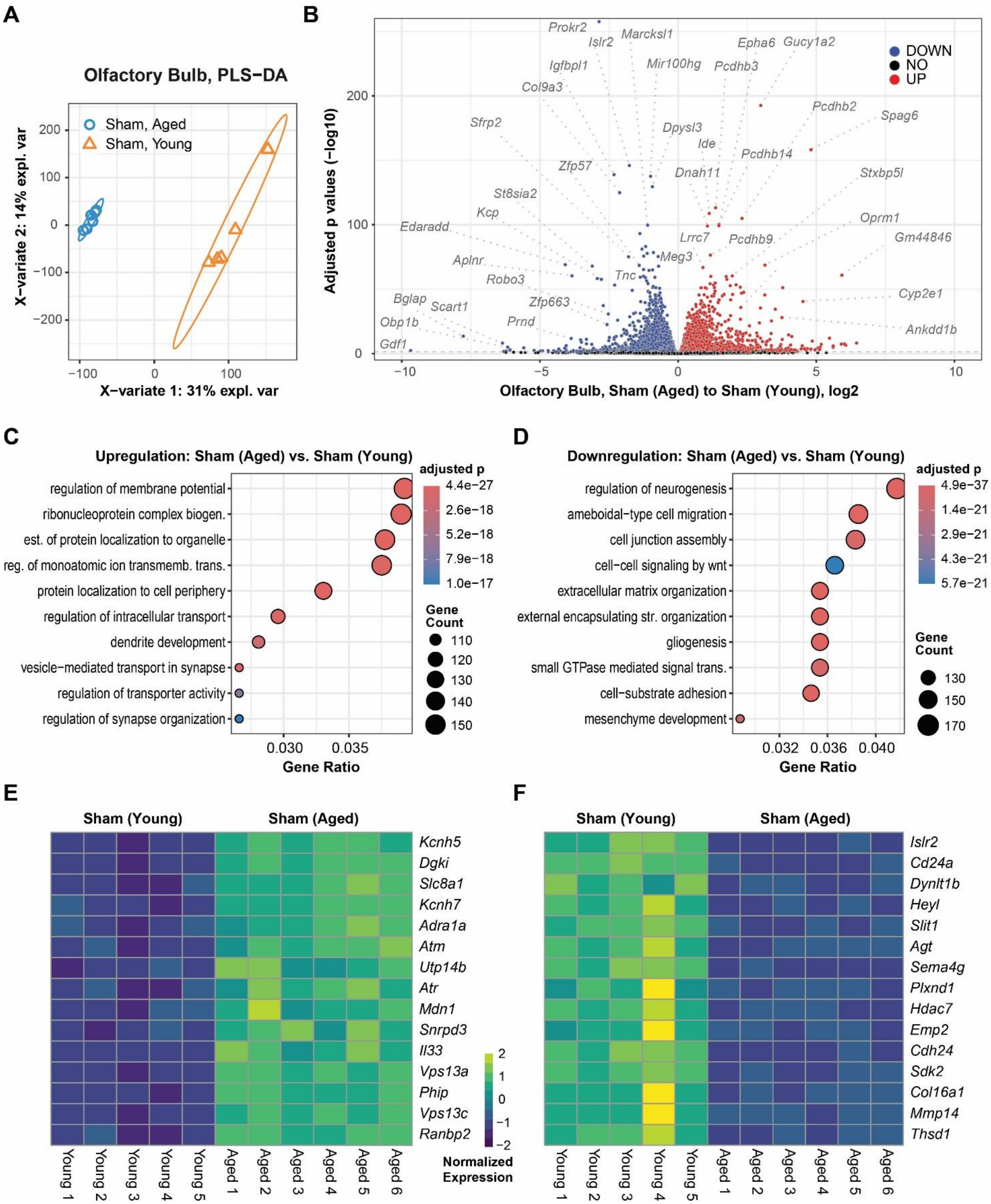

Supplementary Figure 9. **Age-dependent molecular changes in the olfactory bulb (OB) are observed between sham-treated aged and young adult mice. (A)** PLS-DA plot for normalized transcriptome genes shows sample clustering by groups. **(B)** Volcano plot of all genes after pairwise comparison of Aged/Sham vs. Young/Sham. **(C-D)** GO terms pathway enrichment analysis of up- and down-regulated DEGs. **(E-F)** Heatmap of DEGs involved with the top 3 up- and down-regulated genes. n=5 (Young/Sham) and 6 (Aged/Sham) mice.

© 2025. Li Y et al. Published online at http://www.aginganddisease.org/EN/10.14336/AD.2025.0596

SUPPLEMENTARY DATA

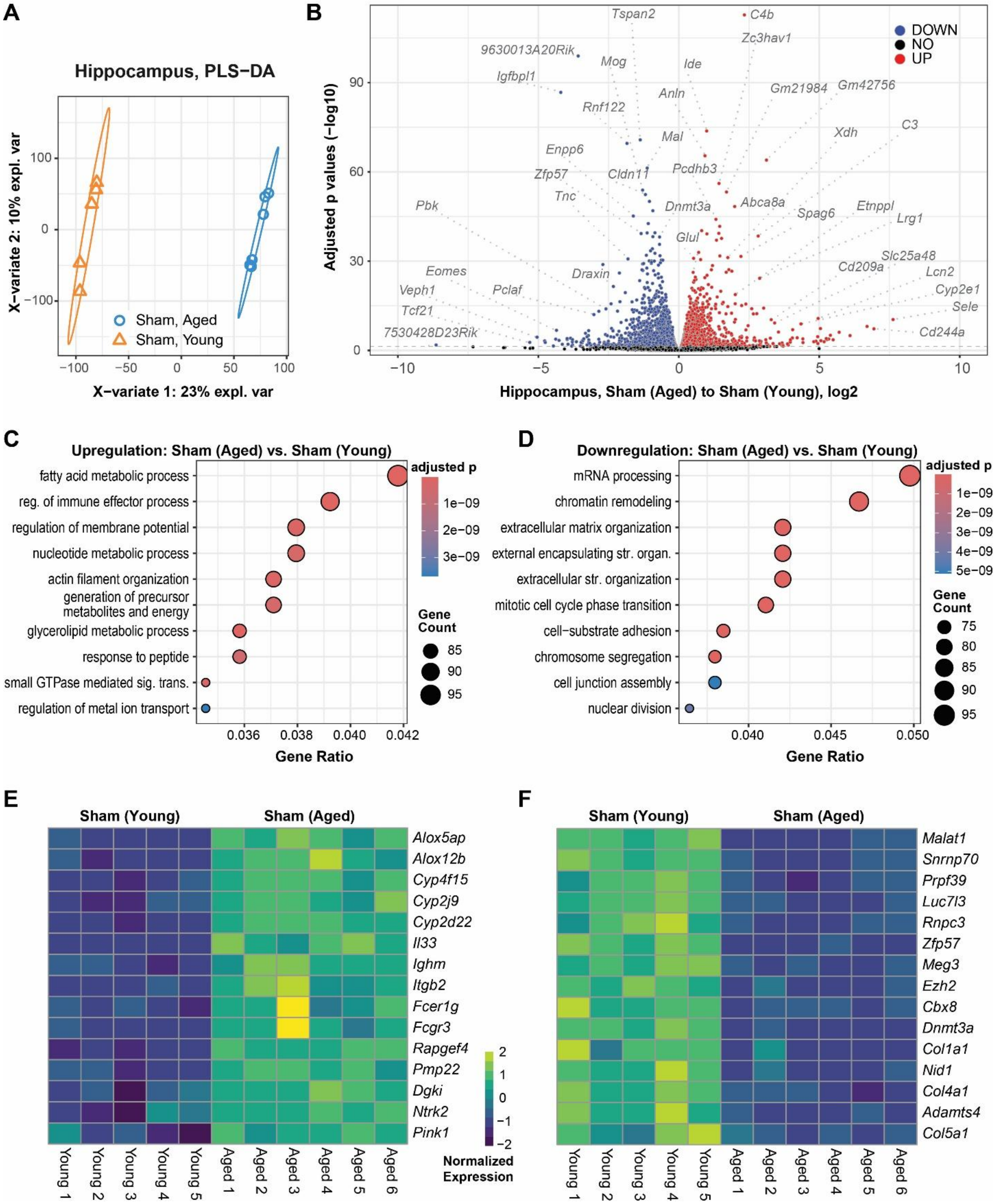

Supplementary Figure 10. **An age-related transcriptomic signature in the hippocampus (HI) is evident when comparing Aged/Sham and Young/Sham mice. (A)** PLS-DA plot for normalized transcriptome genes shows sample clustering by groups. **(B)** Volcano plot of all genes after pairwise comparison of Aged/Sham vs. Young/Sham. **(C-D)** GO terms pathway enrichment analysis of DEGs. **(E-F)** Heatmap of DEGs involved with the top three regulated pathways. n=5 (Young/Sham) and 6 (Aged/Sham).

© 2025. Li Y et al. Published online at http://www.aginganddisease.org/EN/10.14336/AD.2025.0596

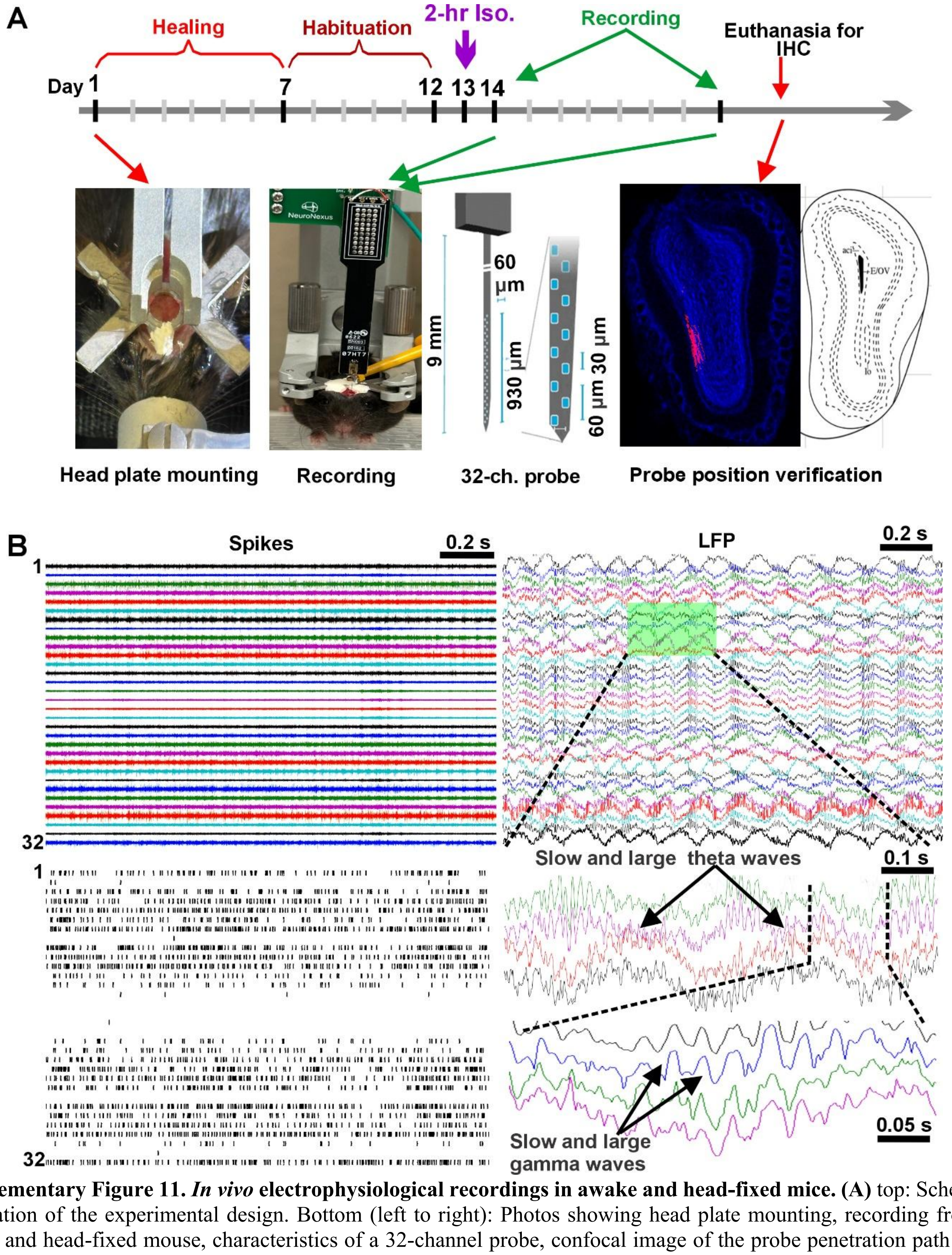

Supplementary Figure 11. ***In vivo*** **electrophysiological recordings in awake and head-fixed mice. (A)** top: Schematic illustration of the experimental design. Bottom (left to right): Photos showing head plate mounting, recording from an awake and head-fixed mouse, characteristics of a 32-channel probe, confocal image of the probe penetration path in the olfactory bulb (OB) mitral cell layer. **(B)** top: Typical traces of spike activities (left) and neural oscillations of local field potential (LFP) (right). Bottom: detected spikes from the top left trace (left) and blown-up traces showing the detection of slow and large theta (top) or fast and small gamma oscillatory waves (bottom). 2-hr: 2 hours, Iso: Isoflurane, IHC: Immunohistochemistry.

© 2025. Li Y et al. Published online at http://www.aginganddisease.org/EN/10.14336/AD.2025.0596
